# Exposure to human-associated fecal indicators and self-reported illness among swimmers at recreational beaches: a cohort study

**DOI:** 10.1186/s12940-017-0308-3

**Published:** 2017-10-02

**Authors:** Melanie D. Napier, Richard Haugland, Charles Poole, Alfred P. Dufour, Jill R. Stewart, David J. Weber, Manju Varma, Jennifer S. Lavender, Timothy J. Wade

**Affiliations:** 1 U.S. Environmental Protection Agency, Office of Research and Development, National Health and Environmental Effects Research Laboratory, 109 T.W. Alexander Drive, Research Triangle Park, NC 27709 USA; 2 Department of Epidemiology, Gillings School of Global Public Health, University of North Carolina-Chapel Hill, 135 Dauer Drive, 2101 McGavran-Greenberg Hall, CB #7435, Chapel Hill, NC 27599 USA; 3 U.S. Environmental Protection Agency, Office of Research and Development, National Exposure Research Laboratory, 26 W. Martin Luther King Drive, Cincinnati, OH USA; 4 Department of Environmental Sciences and Engineering, Gillings School of Global Public Health, University of North Carolina-Chapel Hill, 135 Dauer Drive, 166 Rosenau Hall, CB #7431, Chapel Hill, NC 27599 USA; 5 Division of Infectious Diseases, School of Medicine, University of North Carolina Health Care, Bioinformatics Building, 130 Mason Farm Road, 2nd Floor, CB#7030, Chapel Hill, NC 27599 USA

**Keywords:** Recreational water quality, Fecal indicator bacteria, Gastrointestinal illness, Diarrhea, Respiratory illness, Microbial source tracking, *Bacteroides*

## Abstract

**Background:**

Fecal indicator bacteria used to assess illness risks in recreational waters (e.g., *Escherichia coli*, *Enterococci*) cannot discriminate among pollution sources. To address this limitation, human-associated *Bacteroides* markers have been proposed, but the risk of illness associated with the presence of these markers in recreational waters is unclear. Our objective was to estimate associations between human-associated *Bacteroides* markers in water and self-reported illness among swimmers at 6 U.S. beaches spanning 2003–2007.

**Methods:**

We used data from a prospectively-enrolled cohort of 12,060 swimmers surveyed about beach activities and water exposure on the day of their beach visit. Ten to twelve days later, participants reported gastroinestinal, diarrheal, and respiratory illnesses experienced since the visit. Daily water samples were analyzed for the presence of human-associated *Bacteroides* genetic markers: HF183, BsteriF1, BuniF2, HumM2. We used model-based standardization to estimate risk differences (RD) and 95% confidence intervals (CI). We assessed whether the presence of *Bacteroides* markers were modifiers of the association between general *Enterococcus* and illness among swimmers using interaction contrast.

**Results:**

Overall we observed inconsistent associations between the presence of *Bacteroides* markers and illness. There was a pattern of increased risks of gastrointestinal (RD = 1.9%; 95% CI: 0.1%, 3.7%), diarrheal (RD = 1.3%; 95% CI: -0.2%, 2.7%), and respiratory illnesses (RD = 1.1%; 95% CI: -0.2%, 2.5%) associated with BsteriF1. There was no evidence that *Bacteroides* markers acted as modifiers of *Enterococcus* and illness. Patterns were similar when stratified by water matrix.

**Conclusions:**

Quantitative measures of fecal pollution using *Bacteroides*, rather than presence-absence indicators, may be necessary to accurately assess human risk specific to the presence of human fecal pollution.

**Electronic supplementary material:**

The online version of this article (10.1186/s12940-017-0308-3) contains supplementary material, which is available to authorized users.

## Background

Fecal contamination of waters used for drinking, shellfish harvesting, and recreation is an important public health concern because of possible exposure to a wide range of disease-causing microorganisms. For example, an estimated 170 million enteric and respiratory illnesses worldwide are attributed to swimming in polluted water each year [1]. Water pollution comes from a variety of point (e.g. sewage) and nonpoint (e.g. surface runoff, wildlife, leaky septic systems) sources. Fecal indicator bacteria (FIB) such as *Escherichia coli* (*E. coli*), fecal coliforms, and *Enterococci* are useful for monitoring water quality because their presence has been associated with fecal contamination and swimming-associated illness, usually gastroenteritis [2–4]. These FIB have long been used because they are non-pathogenic, are found in high levels in sewage and feces, and can be correlated with human health effects. However, because they are found in both animal and human feces they cannot be used to distinguish pollution sources [5].

Human fecal contamination is generally considered of greater health risk than fecal contamination from non-human sources due to the high density and range of potentially pathogenic microorgansims that can be found in sewage [6]. In particular, enteric viruses (e.g. Hepatitis A virus, rotavirus, and norovirus), which do not readily transmit infection to a host of a different species [7], are often, but not always [8, 9], believed to be the primary etiologic agent causing swimmer illness in epidemiologic studies of US beaches and outbreaks [10–13]. Thus, water with elevated concentrations of FIB resulting from human sources are more likely to contain human-specific pathogens [7] and pose a greater risk. Swimming in fecally-contamined waters has been associated with self-limiting enteric and respiratory illness but can also lead to more severe illness that results in medical treatment, hospitalization, and/or lost days of school or work [4, 8, 9].

Previous epidemiologic studies that reported an increased risk of gastroenteritis [2, 3, 14], respiratory illness [14, 15], ear ailments [15], or skin illness [14, 16, 17] among swimmers exposed to increasing FIB levels relied on proximity to sewage effluent from wastewater treatment plants as a proxy for human fecal water contamination but lacked water quality measures to confirm the extent to which human fecal contamination impacted the beach on any given day. In recent years, microbial source tracking tools capable of distinguishing human from animal fecal matter have been developed and validated [5]. These tools include both host-associated microbial genetic markers, such as those from the genus *Bacteroides*, and rapid methods, such as quantitative polymerase chain reaction (qPCR), for detection of these markers. *Bacteroides* have been at the forefront of efforts to develop methods that target human sources for a number of reasons [18]: their abundance in the gut of warm-blooded animals [19], the existence of host-specific strains, their high concentrations in sewage [20, 21], their poor survival for long periods in the environment [21], and their persistence during wastewater treatment compared to conventional indicators like *E. coli.* Molecular methods for detection of highly human-associated *Bacteroides* spp. have already been developed and have proven robust [22].

Questions that remain to be answered include whether these markers are associated with human illness and whether they represent an improvement over general, non-specific FIB in terms of characterizing risk. Although a few studies have investigated these relationships, they were limited in size and scope [10, 17, 23, 24]; this paper seeks to address this gap. The primary objective of this study was to estimate the association between four human-associated *Bacteroides* markers and self-reported gastrointestinal (GI), diarrheal, and respiratory illness among swimmers at six U.S. marine and freshwater beaches enrolled in the National Epidemiological and Environmental Assessment of Recreational Water (NEEAR) study from 2003 to 2007. A secondary objective was to determine whether these *Bacteroides* markers modify the association between a general *Enterococcus* indicator and GI and respiratory illnesses.

## Methods

### Study design and participants

The present analysis used data from the NEEAR study, a prospective cohort study conducted to determine relationships between water quality and swimming-associated illnesses. We included 12,060 participants who enrolled in the NEEAR studies at six beaches from 2003 to 2007, met our requirements for body immersion water exposure, and reported incident enteric or respiratory illnesses.

**Fig. 1:**
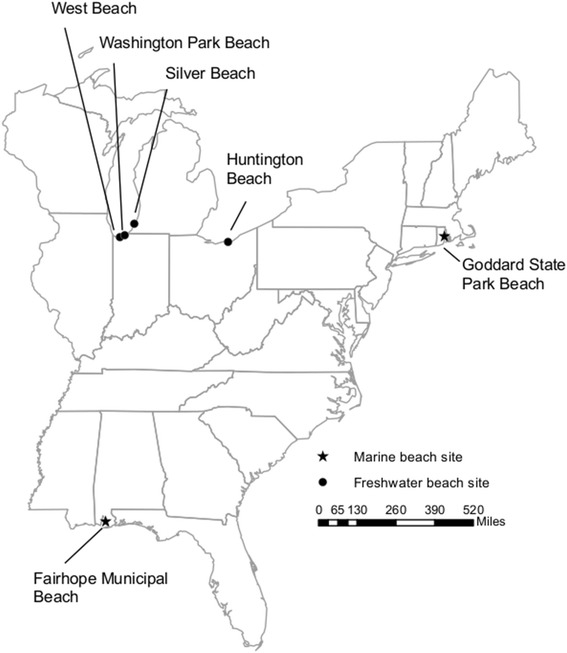
Freshwater and marine beach sites

The beaches were located within 7 miles of treated sewage effluent discharges believed to impact beach fecal contamination. They included four freshwater Great Lakes beaches and two temperate marine beaches (Fig. 1; Table 1). Criteria for beach selection and data collection were described previously [2, 3, 25].

**Table 1:** Beach site characteristics

Type	Beach	Location	Coordinates
Freshwater	Huntington	Lake Erie near Cleveland, Ohio	41°29′30.76″N, 81°55′58.64″W
	Silver	Lake Michigan near St. Joseph, Michigan	42° 6′39.97″N, 86°29′23.37″W
	Washington Park	Lake Michigan in Michigan City, Indiana	41°43′49.84″N, 86°53′46.88″W
	West	Lake Michigan in Portage, Indiana	41°37′35.42″N, 87°12′32.78″W
Marine	Fairhope	Fairhope, Alabama	30°31′37.42″N, 87°54′39.75″W
	Goddard	Near Warwick, Rhode Island	41°40′1.09″N, 71°26′1.85″W

Briefly, adult visitors to the beaches on weekends and holidays in summer completed a survey at enrollment consisting of demographic information and pre-existing illnesses for each household member. Upon departure, these participants answered questions about water exposure (extent, time, duration and location) and other beach activities, such as food and drink consumption and contact with animals. A follow-up telephone interview was completed 10–12 days after the beach visit to determine incident symptoms of gastrointestinal illness, diarrhea, and respiratory illness each household member experienced following the beach visit. Consistent with previous reports [2, 3, 24, 26], “GI illness” referred to any of the following: (1) diarrhea (≥3 loose stools in a 24-h period); (2) vomiting; (3) nausea with stomach ache; or (4) nausea or stomach ache and interference with regular activities (missed time from work/regular activities due to illness). Diarrhea alone was also assessed as a separate outcome. “Respiratory illness” referred to any two of the following: sore throat, cough, runny nose, cold, or fever. Participants were ineligible if they had already completed the study in the previous 28 days, were unaccompanied minors (<18 years), or did not speak English or Spanish.

Because we were interested in illness resulting from exposure to *Bacteroides* markers present in water, we restricted our analysis to participants reporting “body immersion” to the waist or higher (i.e. swimmers). Of the 25,288 NEEAR participants enrolled in the six beaches, 48% (*n* = 12,060) were body immersion swimmers and 36% (*n* = 9091) reported no water contact (i.e. non-swimmers) (Additional file 1: Table S1). Compared to non-swimmers, swimmers were younger (mean age 22.8 years vs. 35.5 years); disproportionately male (48% vs. 37%); disproportionately Hispanic (13% vs. 10%); travelled farther to the beach (mean of 45 miles vs. 38 miles); and reported more sand contact (56% vs. 21%). A quarter of both swimmers and non-swimmers reported having a chronic illness at the beach interview and few (≤6%) reported acute illnesses in the 3 days prior to their beach visits. The amount of missing and incomplete data was negligible (<5%). Participants reporting water contact, but not “body immersion” were excluded from analysis because they comprise a group with heterogeneous water exposure. Other categories of water exposure (i.e. head immersion, swallowed water) were considered in sensitivity analyses described below. Participants who became ill within the 3 days prior to their beach visit were excluded from analysis of the outcome related to their baseline symptoms, but were eligible to be included in analyses of other outcomes.

For the original NEEAR study, procedures, questionnaires, protocols and consent process were reviewed and approved by the Institutional Review Board (IRB) of the Centers for Disease Control and Prevention. NEEAR participants gave verbal informed consent. For the present analysis, IRB exemption was granted by University of North Carolina at Chapel Hill as the dataset was de-identified (# 13–2274).

### *Bacteroides* exposure assessment and analysis

Exposure to human fecal contamination was assessed using archived water samples, in which we determined the concentration of four human-associated *Bacteroides* markers: HF183, BsteriF1, BuniF2, and HumM2. Procedures for water sample collection and filtration for the human-associated *Bacteroides* genetic markers have been described elsewhere [2, 27]. Briefly, water samples were collected three times per day (8:00 AM, 11:00 AM, and 3:00 PM) along three transects perpendicular to the shoreline. At each transect, 1 L of water was collected in waist-high water (1 m deep) and 1 L of water was collected in shin-high water (0.3 m deep) for a total target of 18 samples each day. After collection, samples were maintained on ice at 1–4 °C in coolers for up to 6 h before polycarbonate membrane filtration. The filters were kept at −20 °C, shipped on dry ice to EPA (Cincinnati, OH), and stored at −40 °C for approximately two to 6 years before qPCR analysis. DNA was extracted from the filters by a simple bead milling procedure and aliquots corresponding to two-thirds of the total crude extracts were concentrated 2-fold and purified using a commercially available 96-well silica column based system (DNeasy, Qiagen, Valencia, CA) with binding and elution buffers from another system (DNA-EZ, Gene-Rite, North Brunswick, NJ) as previously described [28].

To determine the concentration of *Bacteroides* markers present in water collected from the beaches, purified DNA extracts were analyzed for *Bacteroides* markers using four qPCR assays. QPCR assays targeting 16S rRNA gene markers of highly human-associated *Bacteroides* species clusters included HF183 TaqMan (hereafter HF183), BsteriF1, and BuniF2 [29] while the HumM2 assay targets a gene encoding a hypothetical protein potentially involved in remodeling surface lipopolysaccharides and polysaccharides in other unidentified, highly human-associated *Bacteroides* species [30]. Among these assays, the HF183 and HumM2 assays have shown the greatest promise for human source tracking due to their high sensitivity in detecting samples that are actually of human origin (e.g. human feces and sewage) as well as their low or nondetectable cross-reactivity with feces from other animals [5, 29–32]. The BsteriF1 and BuniF2 assays have similarly shown high human source sensitivity, but lower specificity due to substantial cross-reactivity with feces from several animal groups including cats and dogs for BsteriF1 and pigs, sheep and chickens for BuniF2 [30, 31]. In addition, total *Bacteroides* genetic markers were also analyzed as indicators of general, nonsource-specific fecal pollution using the GenBac3 qPCR assay [33]. Details of qPCR amplification conditions, including blanks, are described in the Supplement. Out of a total of 2422 initial water samples, 2336 samples passed quality control measures.

We were unable to calculate an average daily concentration for each *Bacteroides* marker due to limited detectable values and prolonged storage of samples, both of which had an unknown effect on the accuracy of the quantifed amount. Instead, we dichotomized exposure as present (detected in ≥2 samples/day) or absent (detected in 0–1 sample/day). Though we lost some detail by dichotomizing this way, it allowed us to maximize our sample size. Dichotomizing exposure conventionally with presence defined as being detected in ≥1 sample/day would have resulted in small numbers over a number of days and therefore unstable effect estimates.

### Confounders

We used directed acyclic graphs [34] (visualized using DAGity [35]) to evaluate potential confounding factors plausibly associated with poor water quality and illness. The final models included age (0–4, 5–11, 12–19, 20–34, ≥35), beach (categorical: Fairhope, Goddard, Huntington, Silver, West, Washington Park), mean number of bathers (continuous), rainfall totals from 3:00 PM the previous day to 8:00 AM on the current day (continuous), sand exposure (binary where 1 = digging in sand or burying body in sand), and, for GI illness and diarrhea, water temperature (continuous)*.* Indicator variables representing beach were included in all models to control for differences in baseline illness among beaches. Robust standard errors were calculated to account for dependence of observations within a household [36].

### Statistical analysis

The association between exposure to each *Bacteroides* marker (HumM2, HF183, BsteriF1, and Bunif2) and self-reported illness was investigated using multivariable regression models adjusted for potential confounders. We used model-based standardization [37–39] to estimate standardized marginal risks, risk differences (RD), and 95% confidence intervals (95% CI) using the total group as the standard. Standard errors for the CI were computed using the delta method [40]. Logistic regression was used to estimate predicted probabilities of the outcome for every value of observed confounders and then combined as a weighted average separately for both levels of the binary exposure. The predicted probabilities were subtracted to produce a marginal estimate of the risk difference comparing *Bacteroides* marker exposure to no exposure. Modification of the *Bacteroides*-illness effect estimates by water matrix (freshwater vs. marine) was assessed by stratification.

To investigate the secondary objective of whether the presence of *Bacteroides* markers of human fecal contamination strengthened the previously-observed association between general, nonsource-specific *Enterococcus* (qPCR Method 1611 [41] and culture Method 1600 [42]) and illness [2, 3], we estimated RD modification with product interactions of *Enterococcus* and *Bacteroides* markers and then assessed for it with an interaction contrast [43]. The interaction contrast is zero when the joint effects of two factors are simply additive [43]. For these analyses, *Enterococcus* was the main effect and the binary *Bacteroides* marker was the modifier. We dichotomized quantitated values of *Enterococcus* according to 2012 EPA recreational water quality guidelines for fresh and marine water. Assuming an illness rate of 36/1000 recreators, the threshold is a geometric mean (GM) of 470 calibrator cell equivalents (CCE)/100 ml for qPCR or a GM of 35 colony forming units (CFU)/100 ml for culture Method 1600. Assuming an illness rate of 32/1000 recreators, the threshold is a GM of 300 CCE/100 ml for qPCR or a GM of 30 CFU/100 ml for culture [44]. *Enterococcus* densities above these guidelines necessitate remedial action, whether beach advisory, closure, or other actions, and so were a practical choice for a binary value. RD modification analyses were also performed with *Enterococcus* coded continuously (average log_10_ count of *Enterococcus* CCE/100 ml per day).

We investigated the robustness of our estimates through sensitivity analyses that tested alternate ways of classifying swimming and *Bacteroides* exposure. First, we repeated our analyses using two additional definitions of swimmer: as participants who reported immersing their head under water, and participants who reported swallowing water. Second, we explored alternate exposure classifications since our primary one did not take into account intensity (i.e. cannot distinguish between situations when *Bacteroides* is detected in 10 vs. 2 samples per day). We therefore explored exposure defined as a count of the number of *Bacteroides* markers detected in ≥2 samples/day: 0 if none of the four markers were detected in ≥2 samples/day, 1 if one of the four markers were detected in ≥2 samples/day, 2 if two of the markers were detected, and so on up to 4. We also explored binary exposure with presence defined conventionally as being detected in ≥1 sample/day.

All analyses were completed using SAS version 9.4 (SAS Institute, Inc., Cary, NC) and Stata version 13 (StataCorp, College Station, TX).

## Results

Descriptive characteristics of NEEAR participants by body immersion status are provided in Additional file 1: Table S1.

**Fig. 2:**
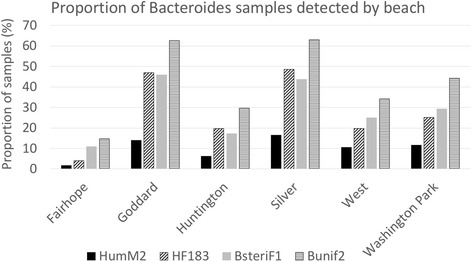
Proportion of *Bacteroides* samples detected by beach

The percent of water samples with human-associated *Bacteroides* markers that were detected, below the limit of detection (undetected), and missing are shown in Table 2 along with the false positive rate for each marker. While human-associated *Bacteroides* markers were detected at every beach, the frequency of markers varied widely by beach and by assay (Fig. 2). Silver and Goddard Beaches had the highest frequencies of detection, regardless of marker, while Fairhope Beach had the lowest. Regardless of beach, BuniF2 and BsteriF1 markers were generally detected more frequently than HF183 and HumM2. For BuniF2, the proportion of detects ranged from 15% (Fairhope) to 63% (Silver) of samples; for BsteriF1, the range was 11% (Fairhope) to 46% (Goddard). HF183 markers were detected in between 4% (Fairhope) and 49% (Silver) of samples. HumM2 markers were detected least often across all beaches, with 2% (Fairhope) to 17% (Silver) of samples testing positive. General, nonspecific *Bacteroides* were detected in neary all (>98%) samples tested using the GenBac3 assay. Overall, the frequency of non-detection among the markers ranged from 58% (BuniF2) to 90% (HumM2) (Table 2).

**Table 2:** Human *Bacteroides* markers detected by qPCR (*n* = 2336 total samples)

Human Marker	Detected in samples N (%)	Nondetect samples N (%)	Missing samples ^a^ N (%)	False positive rate ^b^ (%)
HumM2	233 (10)	2103 (90)	0	0.00
HF183	646 (28)	1690 (72)	0	0.15
BsteriF1	671 (29)	1665 (71)	0	0.20
BuniF2	972 (42)	1364 (58)	0	0.10

Abbreviations: qPCR, quantitative polymerase chain reaction

^a^ Missing out of the 2336 samples that passed quality control measures

^b^ Proportion of samples that tested positive for the assay but were in fact negative

**Fig. 3:**
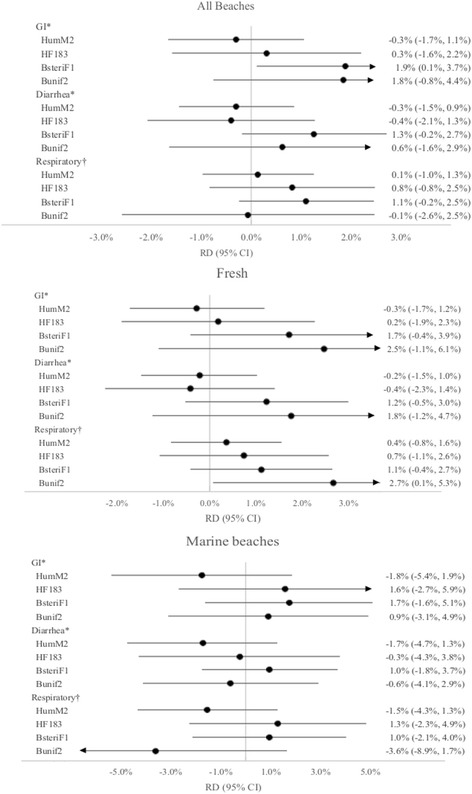
Standardized RD and 95% CI for the association between illness and human-associated *Bacteroides* markers among swimmers. Standardized risk differences (black circles) and 95% confidence intervals (bars) for the association between illness and human-associated *Bacteroides* markers among body immersion swimmers in all beaches (top), freshwater beaches (middle), and marine (bottom) beaches. Arrows show intervals that extend beyond field of vision of diagram. Risk differences compare marker presence (detected in ≥2 samples/day) to absence (detected in 0–1 sample/day) estimated using model-based standardization. *Models for this outcome adjusted for beach, age, mean bathers, sand, rainfall (3 pm the previous day to 8 am present day), and water temperature. †Models for this outcome adjusted for beach, age, mean bathers, sand, and rainfall (3 pm the previous day to 8 am present day). CI, confidence interval; GI, gastrointestinal illness; RD, risk difference

Frequencies and standardized marginal estimates of the RD (95% CI) for illness comparing presence of each *Bacteroides* marker to its absence are shown in Fig. 3 and Additional file 1: Table S2a-c. When the BsteriF1 marker was present, swimmers had approximately 2% more GI illness than when it was absent (RD = 1.9% (0.1%, 3.7%)). Smaller and less precise risks of approximately 1% were observed for diarrhea and respiratory illness (RD = 1.3% (−0.2%, 2.7%) and RD = 1.1% (−0.2%, 2.5%), respectively). Detection of the Bunif2 marker was also associated with an increased risk of GI illness of almost 2% as well (18 per 1000 swimmers), and HF183 with increased risk of respiratory illness by 8 per 1000 swimmers. None of the associations with HumM2 and HF183 were consistently positive. Similar patterns were seen when fresh and marine water were examined separately with the exception of BuniF2. Detection of Bunif2 in freshwater was associated with greater magnitude of risks for all three outcomes: of 25 per 1000, 18 per 1000 and 27 per 1000 swimmers for GI, diarrhea, and respiratory, respectively; for marine beaches, associations with Bunif2 were less precise and closer to the null than fresh water estimates.

Tables 3 and 4 show RDs and 95% CI for the association of *Enterococcus* assessed continuously (by qPCR and culture Method 1600, respectively) and illness modified by each *Bacteroides* marker. As shown previously by Wade et al. [2, 3], we saw a 1.4% and 1.1% increased risk of GI illness and diarrhea with each 1-log_10_ increase in *Enterococcus* measured by qPCR (RD = 1.4% (0.6%, 2.3%) and RD = 1.1% (0.6%, 1.7%), respectively). However, RD estimates did not differ when each *Bacteroides* marker was present vs. absent and interaction contrast estimates were imprecise overall. For one marker (Bunif2) an inverse modification was observed (i.e., the association between *Enterococcus* and illness was attenuated when Bunif2 was present) but no consistent patterns of effect modification were observed. RD estimates for *Enterococcus* assessed dichotomously and illness did not vary by any *Bacteroides* marker (Additional file 1: Table S3–6).

**Table 3:** Modification of the association between *Enterococcus* (CCE/100 ml) exposure^a^ and illness with human-associated *Bacteroides* markers, all beaches

Marker (samples)	Adjusted Risk (%)	Adjusted RD (95% CI)	Interaction Contrast (95% CI)
*Gastrointestional illness* ^b^			
–		1.4% (0.6%, 2.3%)	
HumM2			
0–1	4.4	Ref	
	6.3	1.8% (1.0%, 2.7%)	
≥2	5.9	Ref	
	6.9	1.0% (−0.5%, 2.4%)	−0.9% (−2.3%, 0.6%)
HF183			
0–1	4.4	Ref	
	6.1	1.7% (0.6%, 2.7%)	
≥2	5.7	Ref	
	6.9	1.3% (0.2%, 2.4%)	−0.4% (−1.7%, 0.9%)
BsteriF1			
0–1	7.2	Ref	
	7.2	0.0% (−3.7%, 3.7%)	
≥2	5.6	Ref	
	7.0	1.4% (0.4%, 2.4%)	1.4% (−2.4%, 5.1%)
BuniF2			
0–1	1.1	Ref	
	2.5	1.5% (0.4%, 2.5%)	
≥2	5.7	Ref	
	7.0	1.3% (0.3%, 2.3%)	−0.2% (−1.5%, 1.1%)
*Diarrhea* ^b^			
–		1.1% (0.6%, 1.7%)	
HumM2			
0–1	2.6	Ref	
	4.0	1.4% (0.9%, 1.9%)	
≥2	3.3	Ref	
	4.2	0.9% (0.0%, 1.8%)	−0.5% (−1.4%, 0.4%)
HF183			
0–1	3.6	Ref	
	4.8	1.2% (0.1%, 2.3%)	
≥2	2.7	Ref	
	3.9	1.2% (0.7%, 1.7%)	0.0% (−1.1%, 1.1%)
BsteriF1			
0–1	5.5	Ref	
	5.2	−0.3% (−4.0%, 3.5%)	
≥2	3.0	Ref	
	4.2	1.2% (0.7%, 1.7%)	1.5% (−2.3%, 5.2%)
BuniF2			
0–1	0.9	Ref	
	2.1	1.2% (0.3%, 2.1%)	
≥2	3.2	Ref	
	4.3	1.1% (0.5%, 1.7%)	−0.1% (−1.1%, 0.9%)
*Respiratory Illness* ^c^			
–		0.6% (−0.7%, 1.8%)	
HumM2			
0–1	6.4	Ref	
	6.4	0.0% (−2.0%, 2.0%)	
≥2	4.6	Ref	
	5.6	1.0% (−0.4%, 2.3%)	0.9% (−1.2%, 3.1%)
HF183			
0–1	13.9	Ref	
	9.5	−4.4% (−10.8%, 2.0%)	
≥2	3.9	Ref	
	5.1	1.3% (0.4%, 2.1%)	5.7% (−0.7%, 12%)
BsteriF1			
0–1	8.7	Ref	
	6.7	−1.9% (−7.8%, 4.0)	
≥2	5.7	Ref	
	6.2	0.5% (−0.9%, 1.9%)	2.5% (−3.3%, 8.3%)
BuniF2			
0–1	1.5	Ref	
	2.7	1.2% (0.7%, 1.8%)	
≥2	5.6	Ref	
	6.1	0.5% (−0.9%, 1.8%)	−0.8% (−2.1%, 0.5%)

*Abbreviations*: *CCE* calibrator cell equivalents, *CI* confidence interval, *RD* risk difference, *Ref* reference

^a^ *Enterococcus* exposure measured by qPCR and coded continuously as an average log_10_ count of *Enterococcus* CCE/100 ml per day

^b^ Adjusted for beach, age, mean bathers, sand, rainfall since 3 pm the previous day, water temperature

^c^ Adjusted for beach, age, mean bathers, sand, rainfall since 3 pm the previous day

**Table 4:** Modification of the association between *Enterococcus* (CFU/100 ml) exposure^a^ and illness with human-associated *Bacteroides* markers, all beaches

Marker (samples)	Adjusted risk (%)	Adjusted RD (95% CI)	Interaction contrast (95% CI)
*Gastrointestinal Illness* ^*b*^			
–		−0.1% (−1.5%, 1.2%)	
HumM2			
0–1	8.0	Ref	
	8.4	0.4% (−1.5%, 2.3%)	
≥2	8.7	Ref	
	8.3	−0.3% (−2.1%, 1.4%)	−0.8% (−3.1%, 1.6%)
HF183			
0–1	6.6	Ref	
	8.0	1.4% (−0.5%, 3.2%)	
≥2	10.0	Ref	
	8.8	−1.1% (−3.1%, 0.9%)	−2.5% (−5.1%, 0.2%)
BsteriF1			
0–1	5.3	Ref	
	6.9	1.6% (−0.6%, 3.8%)	
≥2	9.9	Ref	
	8.9	−1.0% (−2.7%, 0.8%)	−2.6% (−5.4%, 0.2%)
Bunif2			
0–1	3.2	Ref	
	6.2	3.1% (0.9%, 5.2%)	
≥2	9.2	Ref	
	8.6	−0.6% (−2.1%, 0.9%)	−3.6% (−6.2%, −1.1%)
*Diarrhea* ^b^			
–		0.2% (−0.8%, 1.2%)	
HumM2			
0–1	4.8	Ref	
	5.6	0.8% (−0.6%, 2.2%)	
≥2	5.4	Ref	
	5.4	0.1% (−1.2%, 1.3%)	−0.7% (−2.5%, 1.0%)
HF183			
0–1	4.7	Ref	
	5.8	1.1% (−0.4%, 2.7%)	
≥2	5.8	Ref	
	5.6	−0.2% (−1.5%, 1.2%)	−1.3% (−3.3%, 0.7%)
BsteriF1			
0–1	3.2	Ref	
	4.7	1.5% (−0.3%, 3.2%)	
≥2	6.3	Ref	
	6.0	−0.4% (−1.7%, 1.0%)	−1.8% (−4.0%, 0.3%)
Bunif2			
0–1	2.1	Ref	
	4.6	2.5% (0.8%, 4.2%)	
≥2	5.8	Ref	
	5.7	−0.1% (−1.2%, 1.0%)	−2.6% (−4.6%, −0.6%)
*Respiratory Illness* ^c^			
–		−0.6% (−2.1%, 0.9%)	
HumM2			
0–1	7.3	Ref	
	6.5	−0.8% (−2.6%, 1.0%)	
≥2	7.4	Ref	
	6.8	−0.6% (−2.7%, 1.5%)	0.2% (−2.3%, 2.7%)
HF183			
0–1	7.6	Ref	
	6.1	−1.4% (−3.7%, 0.9%)	
≥2	7.2	Ref	
	6.8	−0.4% (−2.3%, 1.5%)	1.0% (−2.0%, 3.9%)
BsteriF1			
0–1	5.5	Ref	
	5.3	−0.1% (−2.4%, 2.1%)	
≥2	8.4	Ref	
	7.1	−1.3% (−3.4%, 0.9%)	−1.1% (−4.2%, 2.0%)
Bunif2			
0–1	3.2	Ref	
	5.2	2.1% (0.1%, 4.1%)	
≥2	7.8	Ref	
	6.8	−1.0% (−2.7%, 0.7%)	−3.1% (−5.7%, −0.4%)

*Abbreviations*: *CFU* colony forming units, *CI* confidence interval, *RD* risk difference, *Ref* reference

^a^ *Enterococcus* exposure measured by culture Method 1600 and coded continuously as an average log_10_ count of *Enterococcus* CFU/100 ml per day

^b^ Adjusted for beach, age, mean bathers, sand, rainfall since 3 pm the previous day, water temperature

^c^ Adjusted for beach, age, mean bathers, sand, rainfall since 3 pm the previous day

There was no significant evidence of modification judged by the interaction contrast when analyses were restricted to marine beaches, but there was a pattern of increasing risk of GI illness in the presence vs. absence of HF183, BsteriF1 and BuniF2 when *Enterococcus* was measured by qPCR (Additional file 1: Table S7–8).

Sensitivity analyses for alternate count and binary exposure categorizations and alternate swimmer definitions showed little evidence of association between *Bacteroides* markers and illness. While we found no clear dose-response pattern in which the presence of more *Bacteroides* markers would lead to greater incidence of illness, the greatest risk of illness appeared to occur when 2 or 3 of the four *Bacteroides* markers were detected (Additional file 1: Table S9a-c). Estimates using the more obvious binary categorization of exposure—as 0 (absent) vs. 1 or more samples (present)—were more imprecise and unstable compared to the binary categorization used in our main analysis above. This may be due to the fact that there were few days when HF183 and BuniF2 markers in particular were not detected in any samples (Additional file 1: Fig. S1). When restricted to participants who had immersed their head in water (Additional file 1: Table S10), findings were consistent to those described above but less precise, while findings from participants who swallowed water (Additional file 1: Table S11) were generally farther from the null, and less precise.

## Discussion

In this study, we investigated whether the presence of human-associated *Bacteroides* markers were associated with self-reported GI, diarrheal, and respiratory illness among swimmers in order to determine whether human-associated markers could be useful indicators of fecal contamination at recreational beaches. We report inconsistent associations between the presence of human-associated *Bacteroides* markers and illness, though positive patterns with GI, diarrhea, and respiratory illnesses associated with BsteriF1 detection were observed. We did not detect evidence that *Bacteroides* markers modified the association between the non-source-specific general indicator *Enterococcus* and illness; however in marine waters there was a pattern of an increased GI illness risk in the presence of HF183, BsteriF1 and BuniF2 when *Enterococcus* was measured by qPCR.

Human-associated *Bacteroides* markers were hypothesized to be predictors of swimming-associated illness because of the human pathogens likely in human fecal matter. Of the patterns we identified, positive associations between the BsteriF1 and, to a lesser extent, BuniF2 markers and GI, diarrhea, and respiratory illness are potentially informative. The BsteriF1 and BuniF2 assays have shown high sensitivity in detecting samples that are actually of human origin (e.g. human feces and sewage). However, they also have lower specificity than HF183 and HumM2 due to substantial cross-reactivity with animal feces including cats and dogs for BsteriF1 and pigs, sheep, and chickens for BuniF2 [30, 31]. It is uncertain why these markers performed better than HF183 and HumM2, but one possible reason is that BsteriF1 and Bunif2 were detected more frequently than HF183 and HumM2.

Overall, we observed weaker and more inconsistent associations between illness and *Bacteroides* markers than we expected, especially with HF183 and HumM2. There are several possible explanations for this, related to both biological and uncontrollable factors inherent to the study. The first possibility is that human-associated *Bacteroides* markers may be less persistent than general *Enterococcus* markers in the environment. Several studies have reported that general *Enterococcus* and *Bacteroides* qPCR markers such as Entero1a, GenBac3, and AllBac, persist longer than human-associated microbial source tracking genetic markers, detected by the HF183 [45, 46], HumM2 [46], BacHum [45, 47], and BuniF2 [48] assays. These studies suggest that human-associated markers are most useful as indicators of recent human fecal contamination. If, as suggested by other studies, viral pathogens are also relatively persistent [47, 49], this may explain why health associations were previously established with the general markers, but not among the human associated *Bacteroides* markers examined in this study.

A second possibility is that the densities of the human markers may have been impacted by the long-term freezer storage of the samples at −40 °C. Other analyses have indicated that the densities of *Enterococci* and general *Bacteroides* markers in these archived samples were significantly reduced in comparison to the originally analyzed NEEAR study samples [50]. Because of this, we did not use quantitative estimates of the human markers and instead classified exposure as presence-absence. It is possible this approach reduced the study power to detect associations due to misclassification of exposure (e.g. high exposures were considered the same as low exposures). However, we report results of sensitivity analyses restricted to marine beaches that had a higher correlation between the original and archived sample values of general *Bacteroides* indicator GenBac3, [50], which yielded similar findings to our main analysis.

A third possibility for our findings is that the human-associated *Bacteroides* markers in this study were often undetected. Among the four assays, from 58% to 90% of all samples were negative for the tested *Bacteroides* markers. These factors prompted our use of the presence-absence approach for data analysis, which may have limited our ability to estimate associations, as discussed above. Findings were similar when using a categorical exposure definition in a sensitivity analysis although risk estimates did improve with use of multiple markers. Low target densities and frequencies also contributed to the finding of no association in a previous study of health risks and human-associated markers; in a study of 8797 visitors to a nonpoint source beach in California, Colford et al. [23] concluded that the association between illness and human-specific viruses adenovirus 40, 41 and norovirus could not adequately be evaluated because the viruses were rarely detected.

Finally, it is possible that the level of non-specific fecal contamination may have been so high that the addition of a human marker did not add any additional information to the estimation of illness risk. This analysis was performed at beaches with known human sewage inputs and general *Bacteroides* was detected in >98% of samples, indicating that fecal contamination was present at all beaches throughout the study period. In beaches with lower levels of overall fecal contamination or at sites without a known source of sewage contamination, perhaps human markers would be more informative.

To the best of our knowledge, our study represents the largest effort to date investigating human-associated fecal markers and risk of illness, and the first conducted in settings where disinfected effluent is the primary pollution source. Findings from three smaller previous studies reported no association, though each used assays targeting different human-associated markers and all were conducted at nonpoint source marine beaches. In a prospective cohort study of 5674 people in an urban runoff-impacted California beach, Arnold et al. reported that *Enterococcus faecium* and *Enterococcus faecalis* densities were not consistently associated with swimmer illness [10] using the Scorpion-2 qPCR assay. Similarly, the previously mentioned Colford et al. [23] study of 8797 beachgoers at a different California beach found no association between human-specific viruses and illness, while Sinigalliano et al. [17] found no association with the HF8 and UCD *Bacteroides* markers in a study of 1303 people randomized to water exposure on a South Florida beach impacted by people, dogs, birds, and heavy rainfall. However, in a runoff-impacted study at Doheny beach in California, exposure to human fecal contamination measured by the Scorpion-2 *Enterococcus* qPCR marker was associated with an increased risk of enteric illness in 9525 individuals [24]. Our findings may help inform this limited evidence base of studies investigating human-associated bacterial fecal indicators and human illnesses. The positive patterns of association we found between the BsteriF1 markers and GI, diarrhea, and respiratory illness are novel findings, and may indicate that human markers are indeed associated with illness. Confirming an association necessitates future studies investigating HF183, HumM2, BsteriF1, and BuniF2. Such studies would be strengthened by conducting timely enumeration of *Bacteroides* marker densities by qPCR following water collection. If possible, larger samples may be needed to determine the disease risk of less prevalent outcomes and potentially less abundant human-associated *Bacteroides* markers.

Though few studies have investigated health risks from human-associated fecal indicators, numerous studies have demonstrated an increased risk of GI, diarrheal, respiratory, skin, eye, and ear illnesses among swimmers exposed to elevated general FIB levels [2, 3, 14, 25, 51–53]. These studies demonstrated the value of fecal indicators and many relied on proximity to sewage effluent as a proxy for human presence. Findings have been less consistent for studies in which the predominant contaminants come from nonpoint sources. Some have identified associations between illness and bacteria or viruses [14, 16, 17, 24, 54], while others have not [23, 24]. However it is possible that a human source of fecal contamination may have been nearby even with nonpoint source beaches [14]. A strength of our study is that it did not rely on a proxy; instead, fecal contamination source was directly assessed from the water via the *Bacteroides* markers. Also, because *Bacteroides* are among the most dominant bacteria in the human gut [19], they have been at the forefront of efforts to develop methods that target human sources. This approach may be of particular interest for investigating water bodies impacted by nonpoint sources.

Several issues limit the findings of our study. We relied on measures of daily average water quality as a proxy for an individual swimmer’s exposure. Although these average daily measures may not be indicative of individual exposures, characterizing individual exposures would have been prohibitively difficult. Body immersion swimmers entered the water at multiple time periods and locations and were exposed for varying durations of time (mean duration = 65 min, range: 5–125 min). The study design allowed for the collection of water samples three times a day (8:00 AM, 11:00 AM, and 3:00 PM), at two water depths (shin (0.3 m) and waist height (1.0 m)), and three beach locations to capture the variety of fecal indicator exposures a participant may experience in the water. The design also incorporated water quality measurements over a wide range of study days, so we were able to capture varying water quality conditions. Additionally, a common limitation of this type of large-scale study of water quality is the reliance on self-reported, non-specific symptoms and signs that may have obscured more specific effects of fecal indicators. However, the 10 to 12 day follow-up period reflected the incubation time for likely pathogens that would cause the symptoms of interest. Furthermore, by using self-reported outcomes, we captured the diversity of symptoms potentially associated with recreational water exposure. While the outcomes may have been affected by recall bias, it is unlikely that recall would be differential by varying levels of water quality. In the future, investigators may be able to use non-invasive, objective measures of illness such as multiplex saliva assays to examine the immunoprevalence of pathogens in a population [55].

## Conclusions

Positive patterns with GI illness and diarrhea were noted with the presence of BsteriF1 and BuniF2, but neither these nor other human-associated markers positively improved the association of general indicators already in use at beach sites impacted by sewage effluent. Other human-associated *Bacteroides* markers were inconsistently associated with swimming-associated illness. Quantitative measures, rather than a presence-absence categorization, may more accurately characterize the associations between human markers of fecal contamination and swimming associated illness risk.

## Additional file

Additional file 1: Supplementary material includes methods describing DNA extraction and quantification, and additional tables or figures for main analyses, sensitivity analyses, and modification analyses. (DOCX 251 kb)

## Abbreviations

CCE: Calibrator cell equivalents
CFU: Colony forming units
CI: Confidence interval
*E. coli*: *Escherichia coli*
EPA: ﻿Environmental Protection Agency
FIB: Fecal indicator bacteria
GI: Gastrointestinal
GM: Geometric mean
IRB: Institutional review board
NEEAR: National epidemiological and environmental assessment of recreational water
QPCR: Quantitative polymerase chain reaction
RD: Risk difference
