# Supplementary material for: Exposure to human-associated fecal indicators and self-reported illness among swimmers at recreational beaches: a cohort study
Source: Environ Health. 2017 Oct 2;16:103. doi: 10.1186/s12940-017-0308-3 (PMC5625766; doi:10.1186/s12940-017-0308-3)
Supplement: Supplementary file 1 — Supplementary material includes methods describing DNA extraction and quantification, and additional tables or figures for main analyses, sensitivity analyses, and modification analyses. (DOCX 251 kb) [file 12940_2017_308_MOESM1_ESM.docx]

**Supplemental Material**

**Exposure to human source associated fecal indicators and self-reported illness among swimmers at recreational beaches: a cohort study**

Melanie D. Napier, Richard Haugland, Charles Poole, Alfred P. Dufour, Jill R. Stewart, David J. Weber, Manju Varma, Jennifer S. Lavender, Timothy J. Wade

**Table of Contents**

**Supplemental Methods of DNA extraction and quantification** 3

**Table S1.** Characteristics of NEEAR participants by body immersion status (n=25,288) 4

**Table S2a.** Frequencies and standardized risk differences (95% CI) for the association between illness and human-associated Bacteroides markers among body immersion swimmers in all beaches 6

**Table S2b.** Frequencies and standardized risk differences (95% CI) for the association between illness and human-associated Bacteroides markers among body immersion swimmers in fresh water beaches 7

**Table S2c.** Frequencies and standardized risk differences (95% CI) for the association between illness and human-associated Bacteroides markers among body immersion swimmers in marine beaches 8

**Table S3.** Modification of the adjusted standardized RD (95% CI) for the association between illness and *Enterococcus* qPCR Method 1611 above and below EPA guidelines (geometric mean of 470 CCE/100ml for an illness rate of 36/1000) with detection/non-detection of *Bacteroides* markers among body immersion swimmers in all beaches 9

**Table S4.** Modification of the adjusted standardized RD (95% CI) for the association between illness and *Enterococcus* qPCR Method 1611 above and below EPA guidelines (geometric mean of 300 CCE/100ml for an illness rate of 32/1000) with detection/non-detection of *Bacteroides* markers among body immersion swimmers in all beaches 12

**Table S5.** Modification of the adjusted standardized RD (95% CI) for the association between illness and *Enterococcus* culture Method 1600 above and below EPA guidelines (geometric mean of 35 CFU/100ml for an illness rate of 36/1000) with detection/non-detection of *Bacteroides* markers among body immersion swimmers in all beaches 15

**Table S6.** Modification of the adjusted standardized RD (95% CI) for the association between illness and *Enterococcus* culture Method 1600 above and below EPA guidelines (geometric mean of 30 CFU/100ml for an illness rate of 32/1000) with detection/non-detection of *Bacteroides* markers among body immersion swimmers in all beaches 18

**Table S7 (marine beaches).** Modification of the association between *Enterococcus* (CCE/100ml) exposure^a^ and illness with human-associated *Bacteroides* markers, marine beaches 21

**Table S8 (marine beaches).** Modification of the association between *Enterococcus* (CFU/100ml) exposure^a^ and illness with human-associated *Bacteroides* markers, marine beaches 23

**Table S9a (count, all beaches).** Frequencies and standardized risk differences (95% CI) for the association between illness and number of human-associated Bacteroides markers among body immersion swimmers in all beaches 25

**Table S9b (count, freshwater beaches).** Frequencies and standardized risk differences (95% CI) for the association between illness and number of human-associated *Bacteroides* markers among body immersion swimmers in fresh water beaches 26

**Table S9c (count, marine beaches).** Frequencies and standardized risk differences (95% CI) for the association between illness and number of human-associated *Bacteroides* markers among body immersion swimmers in marine beaches 27

**Figure S1 (0 vs. present in ≥1 samples).** Standardized risk differences and 95% confidence intervals for the association between illness and human-associated *Bacteroides* markers among body immersion swimmers in all, freshwater, and marine beaches. Human-associated indicators categorized as absent vs. present in ≥1 samples/day 28

**Table S10 (head immersion).** Frequencies and standardized risk differences (95% CI) for the association between illness and human-associated *Bacteroides* markers among head immersion swimmers in all beaches 29

**Table S11 (swallowed water).** Frequencies and standardized risk differences (95% CI) for the association between illness and human-associated *Bacteroides* markers among swimmers who swallowed water in all beaches 30

**Supplemental Methods of DNA extraction and quantification**

QPCR amplification was performed using 5 µL of purified DNA extracts in a total reaction volume of 25 µL. Reagent mixes were prepared by combining 12.5 µL of TaqMan® Universal Master Mix (Applied Biosystems, Foster City, CA), 2.5 µL of 2 mg/ml bovine serum albumin, 1 µM of each primer, and 80 nM of probe for each reaction. Amplification occurred with an initial incubation at 50°C for 2 min followed by 95°C for 10 min, then 40 PCR cycles of 95°C for 15 s and 60°C for 1 min. Serial dilutions of commericially prepared plasmid DNA templates (Integrated DNA Technologies, Coralville, IA) containing the amplicons for each assay were analyzed at least in duplicate or more often in triplicate as positive controls in each reaction plate (N = 83 total analyses per dilution per assay). Limits of detection for each assay were based on the estimated plasmid copy number per reaction of the highest dilution of these templates that was detected in >95% of the analyses (~19 copies per reaction). Extracts of blank filters that were prepared in the same manner as the sample extracts were also analyzed as negative controls in each reaction plate. Potential interferences by the sample extracts to the qPCR analyses were assessed by analyses of each extract with a multiplex version of the HF183 assay using an internal amplification control (IAC) template and by analyses with the Sketa22 assay for salmon testes DNA which was added to each sample as a sample processing control (SPC) prior to extraction [1, 2, 3, 4]. Criteria for classifying sample measurements as unacceptable were offset cycle threshold (Ct) values from corresponding control samples of >1.5 and >3.0 for the IAC and SPC assays, respectively, as previously described. All qPCR analyses were performed in an Applied Biosystems StepOnePlus^®^ using the above-mentioned primer and TaqMan™ hybridization probe assays.

**Table S1.** Characteristics of NEEAR participants by body immersion status (n=25,288)

|  | **No water contact** | **Water contact** | |
| --- | --- | --- | --- |
|  |  | **No body immersion** ^a^ | **Body  immersion** |
|  | **(n=9091)** | **(n=4137)** | **(n=12060)** |
|  | **N (%)** | **N (%)** | **N (%)** |
| Age in years, mean (SD), min/max | 35.5 (18), 0/101 | 31.9 (17.6), 0/85 | 22.8 (16.7), 0/103 |
| Age categories, years |  |  |  |
| 0-4 | 503 (6) | 364 (9) | 1218 (10) |
| 5-11 | 390 (4) | 355 (9) | 2953 (25) |
| 12-19 | 911 (10) | 367 (9) | 1848 (16) |
| 20-34 | 2399 (27) | 991 (24) | 2534 (22) |
| 35 and over | 4748 (53) | 1981 (49) | 3182 (27) |
| Missing | 140 | 79 | 325 |
| Sex |  |  |  |
| Male | 3729 (41) | 1511 (37) | 5814 (48) |
| Female | 5356 (59) | 2621 (63) | 6225 (52) |
| Missing | 6 | 5 | 21 |
| Race |  |  |  |
| White | 7266 (80) | 3514 (85) | 9501 (79) |
| Black | 562 (6) | 191 (5) | 518 (4) |
| Asian | 171 (2) | 72 (2) | 140 (1) |
| American Indian | 22 (0) | 17 (0) | 29 (0) |
| Hispanic | 905 (10) | 248 (6) | 1520 (13) |
| Multi-race / other | 148 (2) | 78 (2) | 298 (2) |
| Missing | 17 | 17 | 54 |
| Illnesses in the 3 days prior to beach visit |  |  |  |
| GI illness | 247 (3) | 93 (2) | 221 (2) |
| Vomiting | 94 (1) | 50 (1) | 123 (1) |
| Sore throat | 510 (6) | 227 (5) | 676 (6) |
| Earache | 114 (1) | 39 (1) | 167 (1) |
| Eye irritation | 45 (0) | 22 (1) | 56 (0) |
| Rash | 225 (2) | 89 (2) | 261 (2) |
| Urinary tract infection | 44 (0) | 22 (1) | 49 (0) |
| Any history of chronic GI, skin, respiratory illness or allergies | |  |  |
| No | 6521 (72) | 2943 (71) | 8970 (74) |
| Yes | 2568 (28) | 1192 (29) | 3090 (26) |
| Missing | 2 | 2 | 0 |
| Swam in last week |  |  |  |
| No | 6689 (74) | 2830 (69) | 6844 (57) |
| Yes | 2388 (26) | 1295 (31) | 5198 (43) |
| Missing | 14 | 12 | 18 |
| Miles travelled to beach |  |  |  |
| 0-20 | 5168 (58) | 2114 (52) | 5447 (46) |
| 20-60 | 2531 (28) | 1229 (30) | 4141 (35) |
| 60-100 | 561 (6) | 349 (9) | 1110 (9) |
| >100 | 720 (8) | 408 (10) | 1204 (10) |
| Missing | 111 | 37 | 158 |
| Frequency of travel to beach in summer |  |  |  |
| 0-1 times | 2891 (32) | 1648 (40) | 4166 (35) |
| 2-5 times | 3071 (32) | 1389 (34) | 4483 (37) |
| >5 times | 3110 (34) | 1091 (26) | 3396 (28) |
| Missing | 0 | 0 | 0 |
| Sand contact |  |  |  |
| Dug in sand | 1884 (21) | 1797 (43) | 6662 (55) |
| Buried body in sand | 261 (3) | 267 (6) | 1871 (16) |
| Missing | 0 | 0 | 0 |
| Consumed food |  |  |  |
| No | 4613 (51) | 1764 (43) | 4226 (35) |
| Yes | 4417 (49) | 2348 (57) | 7805 (65) |
| Missing | 61 | 25 | 29 |
| Animal contact 2 days prior to or after beach visit, or between beach visit and phone interview | |  |  |
| No | 2586 (28) | 942 (23) | 2849 (24) |
| Yes | 6505 (72) | 3195 (77) | 9211 (76) |
| Missing | 0 | 0 | 0 |
| All beaches |  |  |  |
| Fairhope | 853 (9) | 340 (8) | 823 (7) |
| Goddard | 1584 (17) | 305 (7) | 1080 (9) |
| Huntington | 1535 (17) | 548 (13) | 757 (6) |
| Silver | 3140 (35) | 1742 (42) | 5372 (45) |
| West | 722 (8) | 475 (11) | 1668 (14) |
| Washington Park | 1257 (14) | 727 (18) | 2360 (20) |

Abbreviatons: NEEAR, National Environmental and Epidemiologic Assessment of Recreational Water study; SD, standard deviation.

^a^ Swimmers were those with body immersion (defined as immersion to the waist or higher). Those without water contact or with water contact but not body immersion were not included in the analysis but are shown in this descriptive table for completeness.

**Table S2a.** Frequencies and standardized risk differences (95% CI) for the association between illness and human-associated Bacteroides markers among body immersion swimmers in all beaches

|  |  | **All beaches** | | | | |
| --- | --- | --- | --- | --- | --- | --- |
|  |  | Cases | N | Crude Risk | Adjusted Risk | Adjusted RD (95% CI) |
| **GI** ^a^ |  |  |  |  |  |  |
| HumM2 | 0-1 samples | 508 | 5712 | 8.9% | 8.5% | Ref |
|  | ≥2 samples | 449 | 5976 | 7.5% | 8.2% | -0.3% (-1.7%, 1.1%) |
| HF183 | 0-1 samples | 254 | 2503 | 10.1% | 8.1% | Ref |
|  | ≥2 samples | 703 | 9185 | 7.7% | 8.4% | 0.3% (-1.6%, 2.2%) |
| BsteriF1 | 0-1 samples | 51 | 575 | 8.9% | 6.8% | Ref |
|  | ≥2 samples | 906 | 11113 | 8.2% | 8.7% | 1.9% (0.1%, 3.7%) |
| Bunif2 | 0-1 samples | 696 | 8453 | 8.2% | 6.6% | Ref |
|  | ≥2 samples | 261 | 3235 | 8.1% | 8.5% | 1.8% (-0.8%, 4.4%) |
| **Diarrhea** ^a^ |  |  |  |  |  |  |
| HumM2 | 0-1 samples | 352 | 5707 | 6.2% | 5.8% | Ref |
|  | ≥2 samples | 287 | 5971 | 4.8% | 5.5% | -0.3% (-1.5%, 0.9%) |
| HF183 | 0-1 samples | 190 | 2501 | 7.6% | 5.9% | Ref |
|  | ≥2 samples | 449 | 9177 | 4.9% | 5.5% | -0.4% (-2.1%, 1.3%) |
| BsteriF1 | 0-1 samples | 38 | 575 | 6.6% | 4.6% | Ref |
|  | ≥2 samples | 601 | 11103 | 5.4% | 5.9% | 1.3% (-0.2%, 2.7%) |
| Bunif2 | 0-1 samples | 462 | 8449 | 5.5% | 5.1% | Ref |
|  | ≥2 samples | 177 | 3229 | 5.5% | 5.7% | 0.6% (-1.6%, 2.9%) |
| **Respiratory** ^b^ |  |  |  |  |  |  |
| HumM2 | 0-1 samples | 335 | 5543 | 6.0% | 5.9% | Ref |
|  | ≥2 samples | 336 | 5790 | 5.8% | 6.0% | 0.1% (-1.0%, 1.3%) |
| HF183 | 0-1 samples | 149 | 2448 | 6.1% | 5.3% | Ref |
|  | ≥2 samples | 522 | 8885 | 5.9% | 6.2% | 0.8% (-0.8%, 2.5%) |
| BsteriF1 | 0-1 samples | 38 | 562 | 6.8% | 5.1% | Ref |
|  | ≥2 samples | 633 | 10771 | 5.9% | 6.2% | 1.1% (-0.2%, 2.5%) |
| Bunif2 | 0-1 samples | 472 | 8202 | 5.8% | 6.0% | Ref |
|  | ≥2 samples | 199 | 3131 | 6.4% | 6.0% | -0.1% (-2.6%, 2.5%) |

^a^ Adjusted for beach, age, mean bathers, sand, rain, water temperature

^b^ Adjusted for beach, age, mean bathers, sand, rain

**Table S2b.** Frequencies and standardized risk differences (95% CI) for the association between illness and human-associated Bacteroides markers among body immersion swimmers in fresh water beaches

|  |  | **Freshwater beaches** | | | | |
| --- | --- | --- | --- | --- | --- | --- |
|  |  | Cases | N | Crude Risk | Adjusted Risk | Adjusted RD (95% CI) |
| **GI** ^a^ |  |  |  |  |  |  |
| HumM2 | 0-1 samples | 403 | 4474 | 9.0% | 8.7% | Ref |
|  | ≥2 samples | 416 | 5373 | 7.7% | 8.4% | -0.3% (-1.7%, 1.2%) |
| HF183 | 0-1 samples | 186 | 1734 | 10.7% | 8.4% | Ref |
|  | ≥2 samples | 633 | 8113 | 7.8% | 8.6% | 0.2% (-1.9%, 2.3%) |
| BsteriF1 | 0-1 samples | 19 | 208 | 9.1% | 7.0% | Ref |
|  | ≥2 samples | 800 | 9639 | 8.3% | 8.7% | 1.7% (-0.4%, 3.9%) |
| Bunif2 | 0-1 samples | 585 | 6925 | 8.4% | 6.1% | Ref |
|  | ≥2 samples | 234 | 2922 | 8.0% | 8.6% | 2.5% (-1.1%, 6.1%) |
| **Diarrhea** ^a^ |  |  |  |  |  |  |
| HumM2 | 0-1 samples | 282 | 4469 | 6.3% | 5.9% | Ref |
|  | ≥2 samples | 265 | 5368 | 4.9% | 5.7% | -0.2% (-1.5%, 1.0%) |
| HF183 | 0-1 samples | 142 | 1732 | 8.2% | 6.1% | Ref |
|  | ≥2 samples | 405 | 8105 | 5.0% | 5.7% | -0.4% (-2.3%, 1.4%) |
| BsteriF1 | 0-1 samples | 13 | 208 | 6.3% | 4.7% | Ref |
|  | ≥2 samples | 534 | 9629 | 5.5% | 6.0% | 1.2% (-0.5%, 3.0%) |
| Bunif2 | 0-1 samples | 388 | 6921 | 5.6% | 4.1% | Ref |
|  | ≥2 samples | 159 | 2916 | 5.5% | 5.8% | 1.8% (-1.2%, 4.7%) |
| **Respiratory** ^b^ |  |  |  |  |  |  |
| HumM2 | 0-1 samples | 253 | 4319 | 5.9% | 5.8% | Ref |
|  | ≥2 samples | 312 | 5197 | 6.0% | 6.2% | 0.4% (-0.8%, 1.6%) |
| HF183 | 0-1 samples | 99 | 1686 | 5.9% | 5.4% | Ref |
|  | ≥2 samples | 466 | 7830 | 6.0% | 6.1% | 0.7% (-1.1%, 2.6%) |
| BsteriF1 | 0-1 samples | 7 | 201 | 3.5% | 5.0% | Ref |
|  | ≥2 samples | 558 | 9315 | 6.0% | 6.1% | 1.1% (-0.4%, 2.7%) |
| Bunif2 | 0-1 samples | 388 | 6700 | 5.8% | 3.4% | Ref |
|  | ≥2 samples | 177 | 2816 | 6.3% | 6.1% | 2.7% (0.1%, 5.3%) |

^a^ Adjusted for beach, age, mean bathers, sand, rain, water temperature

^b^ Adjusted for beach, age, mean bathers, sand, rain

**Table S2c.** Frequencies and standardized risk differences (95% CI) for the association between illness and human-associated Bacteroides markers among body immersion swimmers in marine beaches

|  |  | **Marine beaches** | | | | |
| --- | --- | --- | --- | --- | --- | --- |
|  |  | Cases | N | Crude Risk | Adjusted Risk | Adjusted RD (95% CI) |
| **GI** ^a^ |  |  |  |  |  |  |
| HumM2 | 0-1 samples | 105 | 1238 | 8.5% | 7.9% | Ref |
|  | ≥2 samples | 33 | 603 | 5.5% | 6.1% | -1.8% (-5.4%, 1.9%) |
| HF183 | 0-1 samples | 68 | 769 | 8.8% | 6.7% | Ref |
|  | ≥2 samples | 70 | 1072 | 6.5% | 8.3% | 1.6% (-2.7%, 5.9%) |
| BsteriF1 | 0-1 samples | 32 | 367 | 8.7% | 6.3% | Ref |
|  | ≥2 samples | 106 | 1474 | 7.2% | 8.0% | 1.7% (-1.6%, 5.1%) |
| Bunif2 | 0-1 samples | 111 | 1528 | 7.3% | 6.7% | Ref |
|  | ≥2 samples | 27 | 313 | 8.6% | 7.6% | 0.9% (-3.1%, 4.9%) |
| **Diarrhea** ^a^ |  |  |  |  |  |  |
| HumM2 | 0-1 samples | 70 | 1238 | 5.7% | 5.3% | Ref |
|  | ≥2 samples | 22 | 603 | 3.6% | 3.6% | -1.7% (-4.7%, 1.3%) |
| HF183 | 0-1 samples | 48 | 769 | 6.2% | 5.0% | Ref |
|  | ≥2 samples | 44 | 1072 | 4.1% | 4.7% | -0.3% (-4.3%, 3.8%) |
| BsteriF1 | 0-1 samples | 25 | 367 | 6.8% | 4.3% | Ref |
|  | ≥2 samples | 67 | 1474 | 4.5% | 5.2% | 1.0% (-1.8%, 3.7%) |
| Bunif2 | 0-1 samples | 74 | 1528 | 4.8% | 5.3% | Ref |
|  | ≥2 samples | 18 | 313 | 5.8% | 4.7% | -0.6% (-4.1%, 2.9%) |
| **Respiratory** ^b^ |  |  |  |  |  |  |
| HumM2 | 0-1 samples | 82 | 1224 | 6.7% | 6.3% | Ref |
|  | ≥2 samples | 24 | 593 | 4.0% | 4.7% | -1.5% (-4.3%, 1.3%) |
| HF183 | 0-1 samples | 50 | 762 | 6.6% | 5.3% | Ref |
|  | ≥2 samples | 56 | 1055 | 5.3% | 6.5% | 1.3% (-2.3%, 4.9%) |
| BsteriF1 | 0-1 samples | 31 | 361 | 8.6% | 5.2% | Ref |
|  | ≥2 samples | 75 | 1456 | 5.2% | 6.2% | 1.0% (-2.1%, 4.0%) |
| Bunif2 | 0-1 samples | 84 | 1502 | 5.6% | 8.7% | Ref |
|  | ≥2 samples | 22 | 315 | 7.0% | 5.1% | -3.6% (-8.9%, 1.7%) |

^a^ Adjusted for beach, age, mean bathers, sand, rain, water temperature

^b^ Adjusted for beach, age, mean bathers, sand, rain

**Table S3.** Modification of the adjusted standardized RD (95% CI) for the association between illness and *Enterococcus* qPCR Method 1611 above and below EPA guidelines (geometric mean of 470 CCE/100ml for an illness rate of 36/1000) with detection/non-detection of *Bacteroides* markers among body immersion swimmers in all beaches

| Gastrointestinal Illness | | | | | | | |
| --- | --- | --- | --- | --- | --- | --- | --- |
| Marker (samples) | Enterococcus (CCE/100ml) | Cases | N | Crude Risk (%) | Adjusted Risk (%)^a^ | Adjusted  RD  (95% CI)^a^ | Interaction Contrast  (95% CI) |
| -- | Main association |  |  |  |  | 0.6%  (-2.5%, 3.7%) |  |
| HumM |  |  |  |  |  |  |  |
| 0-1 | <470 | 466 | 5397 | 8.6 | 8.4 | Ref |  |
|  | ≥470 | 42 | 317 | 13.2 | 9.9 | 1.6%  (-2.5%, 5.6%) |  |
| ≥ 2 | <470 | 429 | 5781 | 7.4 | 8.2 | Ref |  |
|  | ≥470 | 20 | 195 | 10.3 | 7.5 | -0.7%  (-4.8%, 3.4%) | **-2.3%**  **(-7.5%, 2.9%)** |
| HF183 |  |  |  |  |  |  |  |
| 0-1 | <470 | 216 | 2231 | 9.7 | 7.8 | Ref |  |
|  | ≥470 | 38 | 274 | 13.9 | 9.6 | 1.8%  (-2.5%, 6.0%) |  |
| ≥ 2 | <470 | 679 | 8947 | 7.6 | 8.5 | Ref |  |
|  | ≥470 | 24 | 238 | 10.1 | 7.7 | -0.8%  (-4.7%, 3.1%) | **-2.5%**  **(-7.9%, 2.8%)** |
| BsteriF1 |  |  |  |  |  |  |  |
| 0-1 | <470 | 153 | 1796 | 8.5 | 7.0 | Ref |  |
|  | ≥470 | 4 | 72 | 5.6 | 3.7 | -3.3%  (-7.1%, 0.4%) |  |
| ≥ 2 | <470 | 742 | 9382 | 7.9 | 8.6 | Ref |  |
|  | ≥470 | 58 | 440 | 13.2 | 9.3 | 0.7%  (-2.8%, 4.2%) | **4.1%**  **(-0.9%, 9.1%)** |
| Bunif2 |  |  |  |  |  |  |  |
| 0-1 | <470 | 50 | 559 | 8.9 | 6.8 | Ref |  |
|  | ≥470 | 1 | 17 | 5.9 | 3.9 | -2.8%  (-9.9%, 4.2%) |  |
| ≥ 2 | <470 | 845 | 10619 | 8.0 | 8.4 | Ref |  |
|  | ≥470 | 61 | 495 | 12.3 | 8.8 | 0.3%  (-2.9%, 3.6%) | **3.2%**  **(-4.6%, 11%)** |

^a^ Adjusted for beach, age, mean bathers, sand, rain, water temperature

**Table S3.** Continued

| **Diarrhea** | | | | | | | |
| --- | --- | --- | --- | --- | --- | --- | --- |
| Marker (samples) | Enterococcus (CCE/100ml) | Cases | N | Crude Risk (%) | Adjusted Risk (%)^a^ | Adjusted  RD  (95% CI) ^a^ | Interaction Contrast  (95% CI) |
| -- | Main association |  |  |  |  | 0.5%  (-2.0%, 3.1%) |  |
| HumM |  |  |  |  |  |  |  |
| 0-1 | <470 | 322 | 5397 | 6.0 | 5.7 | Ref |  |
|  | ≥470 | 30 | 317 | 9.5 | 6.6 | 0.8%  (-2.3%, 4.0%) |  |
| ≥ 2 | <470 | 270 | 5781 | 4.7 | 5.4 | Ref |  |
|  | ≥470 | 17 | 195 | 8.7 | 5.7 | 0.3%  (-3.4%, 3.9%) | **-0.6%**  **(-4.9%, 3.7%)** |
| HF183 |  |  |  |  |  |  |  |
| 0-1 | <470 | 162 | 2231 | 7.3 | 5.8 | Ref |  |
|  | ≥470 | 28 | 274 | 10.2 | 6.7 | 0.9%  (-2.7%, 4.4%) |  |
| ≥ 2 | <470 | 430 | 8947 | 4.8 | 5.5 | Ref |  |
|  | ≥470 | 19 | 238 | 8.0 | 5.7 | 0.2%  (-3.1%, 3.6%) | **-0.6%**  **(-5.1%, 3.8%)** |
| BsteriF1 |  |  |  |  |  |  |  |
| 0-1 | <470 | 104 | 1796 | 5.8 | 4.8 | Ref |  |
|  | ≥470 | 2 | 72 | 2.8 | 1.9 | -2.8%  (-5.8%, 0.1%) |  |
| ≥ 2 | <470 | 488 | 9382 | 5.2 | 5.8 | Ref |  |
|  | ≥470 | 45 | 440 | 10.2 | 6.5 | 0.7%  (-2.1%, 3.5%) | **3.5%**  **(-0.5%, 7.6%)** |
| Bunif2 |  |  |  |  |  |  |  |
| 0-1 | <470 | 38 | 559 | 6.8 | 5.3 | Ref |  |
|  | ≥470 | 0 | 17 | 0.0 | 0.0 | NA |  |
| ≥ 2 | <470 | 554 | 10619 | 5.2 | 5.6 | Ref |  |
|  | ≥470 | 47 | 495 | 9.5 | 6.3 | 0.7%  (-2.1%, 3.4%) | **NA** |

NA, not able to estimated

^a^ Adjusted for beach, age, mean bathers, sand, rain, water temperature

**Table S3.** Continued

| **Respiratory** | | | | | | | |
| --- | --- | --- | --- | --- | --- | --- | --- |
| Marker (samples) | Enterococcus (CCE/100ml) | Cases | N | Crude Risk (%) | Adjusted Risk (%)^a^ | Adjusted  RD  (95% CI) ^a^ | Interaction Contrast  (95% CI) |
| -- | Main association |  |  |  |  | -1.3%  (-4.0%, 1.4%) |  |
| HumM |  |  |  |  |  |  |  |
| 0-1 | <470 | 321 | 5236 | 6.1 | 5.9 | Ref |  |
|  | ≥470 | 14 | 309 | 4.5 | 4.8 | -1.1%  (-4.2%, 2.0%) |  |
| ≥ 2 | <470 | 328 | 5603 | 5.9 | 6.1 | Ref |  |
|  | ≥470 | 8 | 187 | 4.3 | 4.5 | -1.7%  (-6.4%, 3.1%) | **-0.5%**  **(-6.1%, 5.1%)** |
| HF183 |  |  |  |  |  |  |  |
| 0-1 | <470 | 141 | 2182 | 6.5 | 5.6 | Ref |  |
|  | ≥470 | 8 | 268 | 3.0 | 3.0 | -2.6%  (-5.3%, 0.0%) |  |
| ≥ 2 | <470 | 508 | 8657 | 5.9 | 6.2 | Ref |  |
|  | ≥470 | 14 | 228 | 6.1 | 6.3 | 0.1%  (-4.5%, 4.8%) | **2.8%**  **(-2.7%, 8.3%)** |
| BsteriF1 |  |  |  |  |  |  |  |
| 0-1 | <470 | 97 | 1726 | 5.6 | 5.1 | Ref |  |
|  | ≥470 | 2 | 68 | 2.9 | 2.5 | -2.6%  (-6.2%, 1.0%) |  |
| ≥ 2 | <470 | 552 | 9113 | 6.1 | 6.3 | Ref |  |
|  | ≥470 | 20 | 428 | 4.7 | 4.8 | -1.4%  (-4.4%, 1.6%) | **1.2%**  **(-3.4%, 5.8%)** |
| Bunif2 |  |  |  |  |  |  |  |
| 0-1 | <470 | 38 | 546 | 7.0 | 6.0 | Ref |  |
|  | ≥470 | 0 | 17 | 0.0 | 0.0 | NA |  |
| ≥ 2 | <470 | 611 | 10293 | 5.9 | 6.0 | Ref |  |
|  | ≥470 | 22 | 479 | 4.6 | 4.9 | -1.1%  (-4.0%, 1.8%) | **NA** |

NA, not able to estimated

^a^ Adjusted for beach, age, mean bathers, sand, rain

**Table S4.** Modification of the adjusted standardized RD (95% CI) for the association between illness and *Enterococcus* qPCR Method 1611 above and below EPA guidelines (geometric mean of 300 CCE/100ml for an illness rate of 32/1000) with detection/non-detection of *Bacteroides* markers among body immersion swimmers in all beaches

| **Gastrointestinal Illness** | | | | | | | |
| --- | --- | --- | --- | --- | --- | --- | --- |
| Marker (samples) | Enterococcus (CCE/100ml) | Cases | N | Crude Risk (%) | Adjusted Risk (%)^a^ | Adjusted  RD  (95% CI) ^a^ | Interaction Contrast  (95% CI) |
| -- | Main association |  |  |  |  | 1.6%  (-1.0%, 4.3%) |  |
| HumM |  |  |  |  |  |  |  |
| 0-1 | <300 | 429 | 5104 | 5.8 | 8.2 | Ref |  |
|  | ≥300 | 79 | 610 | 6.7 | 10.8 | 2.6%  (-0.6%, 5.8%) |  |
| ≥ 2 | <300 | 428 | 5752 | 5.7 | 8.1 | Ref |  |
|  | ≥300 | 21 | 224 | 4.5 | 7.5 | -0.7%  (-4.6%, 3.2%) | **-3.3%**  **(-7.9%, 1.4%)** |
| HF183 |  |  |  |  |  |  |  |
| 0-1 | <300 | 195 | 2048 | 6.1 | 7.8 | Ref |  |
|  | ≥300 | 59 | 457 | 5.5 | 10.3 | 2.6%  (-1.1%, 6.3%) |  |
| ≥ 2 | <300 | 662 | 8808 | 5.6 | 8.3 | Ref |  |
|  | ≥300 | 41 | 377 | 6.9 | 8.8 | 0.4%  (-3.1%, 3.9%) | **-2.1%**  **(-7%, 2.7%)** |
| BsteriF1 |  |  |  |  |  |  |  |
| 0-1 | <300 | 131 | 1643 | 4.9 | 6.5 | Ref |  |
|  | ≥300 | 26 | 225 | 8.0 | 9.3 | 2.8%  (-1.9%, 7.4%) |  |
| ≥ 2 | <300 | 726 | 9213 | 5.9 | 8.6 | Ref |  |
|  | ≥300 | 74 | 609 | 5.4 | 9.1 | 0.5%  (-2.4%, 3.5%) | **-2.2%**  **(-7.6%, 3.1%)** |
| Bunif2 |  |  |  |  |  |  |  |
| 0-1 | <300 | 33 | 444 | 5.9 | 5.6 | Ref |  |
|  | ≥300 | 18 | 132 | 9.1 | 12.0 | 6.4%  (-0.6%, 13.3%) |  |
| ≥ 2 | <300 | 824 | 10412 | 5.7 | 8.4 | Ref |  |
|  | ≥300 | 82 | 702 | 5.6 | 8.8 | 0.3%  (-2.4%, 3.1%) | **-6.0%**  **(-13.4%, 1.3%)** |

^a^ Adjusted for beach, age, mean bathers, sand, rain, water temperature

**Table S4.** Continued

| **Diarrhea** | | | | | | | |
| --- | --- | --- | --- | --- | --- | --- | --- |
| Marker (samples) | Enterococcus (CCE/100ml) | Cases | N | Crude Risk (%) | Adjusted Risk (%)^a^ | Adjusted  RD (95% CI) ^a^ | Interaction Contrast  (95% CI) |
| -- | Main association |  |  |  |  | 2.0%  (-0.4%, 4.3%) |  |
| HumM |  |  |  |  |  |  |  |
| 0-1 | <300 | 291 | 5104 | 5.7 | 5.5 | Ref |  |
|  | ≥300 | 61 | 610 | 10.0 | 8.2 | 2.6%  (-0.2%, 5.4%) |  |
| ≥ 2 | <300 | 269 | 5752 | 4.7 | 5.3 | Ref |  |
|  | ≥300 | 18 | 224 | 8.0 | 6.0 | 0.6%  (-3.0%, 4.2%) | **-2.0%**  **(-6.2%, 2.1%)** |
| HF183 |  |  |  |  |  |  |  |
| 0-1 | <300 | 144 | 2048 | 7.0 | 5.7 | Ref |  |
|  | ≥300 | 46 | 457 | 10.1 | 8.4 | 2.7%  (-0.8%, 6.1%) |  |
| ≥ 2 | <300 | 416 | 8808 | 4.7 | 5.3 | Ref |  |
|  | ≥300 | 33 | 377 | 8.8 | 6.9 | 1.6%  (-1.6%, 4.7%) | **-1.1%**  **(-5.5%, 3.2%)** |
| BsteriF1 |  |  |  |  |  |  |  |
| 0-1 | <300 | 87 | 1643 | 5.3 | 4.4 | Ref |  |
|  | ≥300 | 19 | 225 | 8.4 | 7.4 | 3.0%  (-1.3%, 7.3%) |  |
| ≥ 2 | <300 | 473 | 9213 | 5.1 | 5.7 | Ref |  |
|  | ≥300 | 60 | 609 | 9.9 | 6.9 | 1.2%  (-1.4%, 3.8%) | **-1.8%**  **(-6.5%, 3.0%)** |
| Bunif2 |  |  |  |  |  |  |  |
| 0-1 | <300 | 25 | 444 | 5.6 | 4.5 | Ref |  |
|  | ≥300 | 13 | 132 | 9.8 | 10.2 | 5.7%  (-1.6%, 13.0%) |  |
| ≥ 2 | <300 | 535 | 10412 | 5.1 | 5.5 | Ref |  |
|  | ≥300 | 66 | 702 | 9.4 | 6.8 | 1.3%  (-1.2%, 3.8%) | **-4.4%**  **(-12.1%, 3.3%)** |

NA, not able to estimated

^a^ Adjusted for beach, age, mean bathers, sand, rain, water temperature

**Table S4.** Continued

| **Respiratory Illness** | | | | | | | |
| --- | --- | --- | --- | --- | --- | --- | --- |
| Marker (samples) | Enterococcus (CCE/100ml) | Cases | N | Crude Risk (%) | Adjusted Risk (%)^a^ | Adjusted  RD  (95% CI) ^a^ | Interaction Contrast  (95% CI) |
| -- | Main association |  |  |  |  | 0.2%  (-2.1%, 2.6%) |  |
| HumM |  |  |  |  |  |  |  |
| 0-1 | <300 | 294 | 4947 | 5.9 | 5.8 | Ref |  |
|  | ≥300 | 41 | 598 | 6.9 | 6.5 | 0.6%  (-1.9%, 3.2%) |  |
| ≥ 2 | <300 | 326 | 5572 | 5.9 | 6.1 | Ref |  |
|  | ≥300 | 10 | 218 | 4.6 | 5.1 | -0.9%  (-5.5%, 3.7%) | **-1.6%**  **(-6.6%, 3.4%)** |
| HF183 |  |  |  |  |  |  |  |
| 0-1 | <300 | 124 | 2001 | 6.2 | 5.5 | Ref |  |
|  | ≥300 | 25 | 449 | 5.6 | 5.0 | -0.5%  (-3.1%, 2.1%) |  |
| ≥ 2 | <300 | 496 | 8518 | 5.8 | 6.1 | Ref |  |
|  | ≥300 | 26 | 367 | 7.1 | 6.9 | 0.8%  (-3.0%, 4.6%) | **1.3%**  **(-3.3%, 5.9%)** |
| BsteriF1 |  |  |  |  |  |  |  |
| 0-1 | <300 | 81 | 1577 | 5.1 | 4.8 | Ref |  |
|  | ≥300 | 18 | 217 | 8.3 | 6.7 | 1.9%  (-2.2%, 5.9%) |  |
| ≥ 2 | <300 | 539 | 8942 | 6.0 | 6.2 | Ref |  |
|  | ≥300 | 33 | 599 | 5.5 | 5.5 | -0.8%  (-3.3%, 1.8%) | **-2.6%**  **(-7.2%, 1.9%)** |
| Bunif2 |  |  |  |  |  |  |  |
| 0-1 | <300 | 26 | 435 | 6.0 | 5.5 | Ref |  |
|  | ≥300 | 12 | 128 | 9.4 | 6.0 | 2.3%  (-3.6%, 8.2%) |  |
| ≥ 2 | <300 | 594 | 10084 | 5.9 | 6.0 | Ref |  |
|  | ≥300 | 39 | 688 | 5.7 | 5.8 | -0.2%  (-2.7%, 2.3%) | **-2.5%**  **(-8.7%, 3.8%)** |

NA, not able to estimated

^a^ Adjusted for beach, age, mean bathers, sand, rain

**Table S5.** Modification of the adjusted standardized RD (95% CI) for the association between illness and *Enterococcus* culture Method 1600 above and below EPA guidelines (geometric mean of 35 CFU/100ml for an illness rate of 36/1000) with detection/non-detection of *Bacteroides* markers among body immersion swimmers in all beaches

| **Gastrointestinal Illness** | | | | | | | |
| --- | --- | --- | --- | --- | --- | --- | --- |
| Marker (samples) | Enterococcus (CFU/100ml) | Cases | N | Crude Risk (%) | Adjusted Risk (%)^a^ | Adjusted  RD  (95% CI) ^a^ | Interaction Contrast  (95% CI) |
| -- | Main association |  |  |  |  | 0.8%  (-0.6%, 2.1%) |  |
| HumM |  |  |  |  |  |  |  |
| 0-1 | <35 | 375 | 4438 | 8.4 | 8.2 | Ref |  |
|  | ≥35 | 133 | 1276 | 10.4 | 9.7 | 1.5%  (-0.6%, 3.6%) |  |
| ≥ 2 | <35 | 268 | 3620 | 7.4 | 8.0 | Ref |  |
|  | ≥35 | 181 | 2356 | 7.7 | 8.4 | 0.4%  (-1.4%, 2.2%) | **-1.1%**  **(-3.8%, 1.6%)** |
| HF183 |  |  |  |  |  |  |  |
| 0-1 | <35 | 177 | 1918 | 9.2 | 7.5 | Ref |  |
|  | ≥35 | 77 | 587 | 13.1 | 10.2 | 2.7%  (-0.3%, 5.7%) |  |
| ≥ 2 | <35 | 466 | 6140 | 7.6 | 8.4 | Ref |  |
|  | ≥35 | 237 | 3045 | 7.8 | 8.5 | 0.1%  (-1.4%, 1.7%) | **-2.6%**  **(-5.9%, 0.8%)** |
| BsteriF1 |  |  |  |  |  |  |  |
| 0-1 | <35 | 131 | 1641 | 8.0 | 6.3 | Ref |  |
|  | ≥35 | 26 | 227 | 11.5 | 9.8 | 3.4%  (-1.6%, 8.5%) |  |
| ≥ 2 | <35 | 512 | 6417 | 8.0 | 8.6 | Ref |  |
|  | ≥35 | 288 | 3405 | 8.5 | 8.8 | 0.3%  (-1.2%, 1.7%) | **-3.2%**  **(-8.4%, 2.1%)** |
| Bunif2 |  |  |  |  |  |  |  |
| 0-1 | <35 | 29 | 414 | 7.0 | 5.0 | Ref |  |
|  | ≥35 | 22 | 162 | 13.6 | 12.3 | 7.3%  (0.3%, 14.2%) |  |
| ≥ 2 | <35 | 614 | 7644 | 8.0 | 8.3 | Ref |  |
|  | ≥35 | 292 | 3470 | 8.4 | 8.7 | 0.3%  (-1.0%, 1.7%) | **-6.9%**  **(-14.0%, 0.2%)** |

^a^ Adjusted for beach, age, mean bathers, sand, rain, water temperature

**Table S5.** Continued

| **Diarrhea** | | | | | | | |
| --- | --- | --- | --- | --- | --- | --- | --- |
| Marker (samples) | Enterococcus (CFU/100ml) | Cases | N | Crude Risk (%) | Adjusted Risk (%)^a^ | Adjusted  RD (95% CI) ^a^ | Interaction Contrast  (95% CI) |
| -- | Main association |  |  |  |  | 0.9%  (-0.3%, 2.0%) |  |
| HumM |  |  |  |  |  |  |  |
| 0-1 | <35 | 253 | 4438 | 5.7 | 5.4 | Ref |  |
|  | ≥35 | 99 | 1276 | 7.8 | 7.1 | 1.6%  (-0.2%, 3.5%) |  |
| ≥ 2 | <35 | 171 | 3620 | 4.7 | 5.3 | Ref |  |
|  | ≥35 | 116 | 2356 | 4.9 | 5.7 | 0.5%  (-1.1%, 2.0%) | **-1.2%**  **(-3.5%, 1.2%)** |
| HF183 |  |  |  |  |  |  |  |
| 0-1 | <35 | 131 | 1918 | 6.8 | 5.4 | Ref |  |
|  | ≥35 | 59 | 587 | 10.1 | 7.4 | 2.0%  (-0.6%, 4.5%) |  |
| ≥ 2 | <35 | 293 | 6140 | 4.8 | 5.4 | Ref |  |
|  | ≥35 | 156 | 3045 | 5.1 | 5.9 | 0.5%  (-0.8%, 1.8%) | **-1.4%**  **(-4.3%, 1.5%)** |
| BsteriF1 |  |  |  |  |  |  |  |
| 0-1 | <35 | 88 | 1641 | 5.4 | 4.3 | Ref |  |
|  | ≥35 | 18 | 227 | 7.9 | 6.8 | 2.5%  (-1.9%, 7.0%) |  |
| ≥ 2 | <35 | 336 | 6417 | 5.2 | 5.7 | Ref |  |
|  | ≥35 | 197 | 3405 | 5.8 | 6.2 | 0.5%  (-0.7%, 1.8%) | **-2.0%**  **(-6.6%, 2.6%)** |
| Bunif2 |  |  |  |  |  |  |  |
| 0-1 | <35 | 21 | 414 | 5.1 | 3.7 | Ref |  |
|  | ≥35 | 17 | 162 | 10.5 | 9.6 | 5.9%  (-0.6%, 12.3%) |  |
| ≥ 2 | <35 | 403 | 7644 | 5.3 | 5.5 | Ref |  |
|  | ≥35 | 198 | 3470 | 5.7 | 6.0 | 0.5%  (-0.6%, 1.7%) | **-5.3%**  **(-11.9%, 1.2%)** |

NA, not able to estimated

^a^ Adjusted for beach, age, mean bathers, sand, rain, water temperature

**Table S5.** Continued

| **Respiratory Illness** | | | | | | | |
| --- | --- | --- | --- | --- | --- | --- | --- |
| Marker (samples) | Enterococcus (CFU/100ml) | Cases | N | Crude Risk (%) | Adjusted Risk (%)^a^ | Adjusted  RD  (95% CI) ^a^ | Interaction Contrast  (95% CI) |
| -- | Main association |  |  |  |  | -1.0%  (-2.1%, 0.2%) |  |
| HumM |  |  |  |  |  |  |  |
| 0-1 | <35 | 269 | 4299 | 6.3 | 6.2 | Ref |  |
|  | ≥35 | 66 | 1246 | 5.3 | 5.0 | -1.1%  (-2.7%, 0.5%) |  |
| ≥ 2 | <35 | 208 | 3511 | 5.9 | 6.5 | Ref |  |
|  | ≥35 | 128 | 2279 | 5.6 | 5.5 | -1.0%  (-2.5%, 0.6%) | **0.2%**  **(-2.0%, 2.4%)** |
| HF183 |  |  |  |  |  |  |  |
| 0-1 | <35 | 124 | 1867 | 6.6 | 5.8 | Ref |  |
|  | ≥35 | 25 | 583 | 4.3 | 3.7 | -2.1%  (-4.3%, 0.1%) |  |
| ≥ 2 | <35 | 353 | 5943 | 5.9 | 6.4 | Ref |  |
|  | ≥35 | 169 | 2942 | 5.7 | 5.7 | -0.7%  (-2.0%, 0.6%) | **1.4%**  **(-1.2%, 3.9%)** |
| BsteriF1 |  |  |  |  |  |  |  |
| 0-1 | <35 | 81 | 1569 | 5.2 | 4.9 | Ref |  |
|  | ≥35 | 18 | 225 | 8.0 | 6.8 | 1.9%  (-2.3%, 6.1%) |  |
| ≥ 2 | <35 | 396 | 6241 | 6.3 | 6.7 | Ref |  |
|  | ≥35 | 176 | 3300 | 5.3 | 5.2 | -1.5%  (-2.7%, -0.3%) | **-3.4%**  **(-7.8%, 1.0%)** |
| Bunif2 |  |  |  |  |  |  |  |
| 0-1 | <35 | 23 | 401 | 5.7 | 5.3 | Ref |  |
|  | ≥35 | 15 | 162 | 9.3 | 8.6 | 3.3%  (-2.9%, 9.6%) |  |
| ≥ 2 | <35 | 454 | 7409 | 6.1 | 6.4 | Ref |  |
|  | ≥35 | 179 | 3363 | 5.3 | 5.1 | -1.2%  (-2.4%, -0.1%) | **-4.5%**  **(-11.0%, 1.9%)** |

NA, not able to estimated

^a^ Adjusted for beach, age, mean bathers, sand, rain

**Table S6.** Modification of the adjusted standardized RD (95% CI) for the association between illness and *Enterococcus* culture Method 1600 above and below EPA guidelines (geometric mean of 30 CFU/100ml for an illness rate of 32/1000) with detection/non-detection of *Bacteroides* markers among body immersion swimmers in all beaches

| **Gastrointestinal Illness** | | | | | | | |
| --- | --- | --- | --- | --- | --- | --- | --- |
| Marker (samples) | Enterococcus (CFU/100ml) | Cases | N | Crude Risk (%) | Adjusted Risk (%)^a^ | Adjusted  RD  (95% CI) ^a^ | Interaction Contrast  (95% CI) |
| -- | Main association |  |  |  |  | 0.6%  (-0.7%, 2.0%) |  |
| HumM |  |  |  |  |  |  |  |
| 0-1 | <30 | 368 | 4316 | 8.5 | 8.3 | Ref |  |
|  | ≥30 | 140 | 1398 | 10.0 | 9.2 | 0.9%  (-1.1%, 3.0%) |  |
| ≥ 2 | <30 | 263 | 3583 | 7.3 | 7.9 | Ref |  |
|  | ≥30 | 186 | 2393 | 7.8 | 8.5 | 0.6%  (-1.3%, 2.4%) | **-0.4%**  **(-3.0%, 2.3%)** |
| HF183 |  |  |  |  |  |  |  |
| 0-1 | <30 | 175 | 1888 | 9.3 | 7.5 | Ref |  |
|  | ≥30 | 79 | 617 | 12.8 | 9.8 | 2.3%  (-0.6%, 5.3%) |  |
| ≥ 2 | <30 | 456 | 6011 | 7.6 | 8.4 | Ref |  |
|  | ≥30 | 247 | 3174 | 7.8 | 8.5 | 0.1% (-1.4%, 1.6%) | **-2.2%**  **(-5.5%, 1.0%)** |
| BsteriF1 |  |  |  |  |  |  |  |
| 0-1 | <30 | 129 | 1611 | 8.0 | 6.4 | Ref |  |
|  | ≥30 | 28 | 257 | 10.9 | 8.8 | 2.4%  (-1.9%, 6.7%) |  |
| ≥ 2 | <30 | 502 | 6288 | 8.0 | 8.6 | Ref |  |
|  | ≥30 | 298 | 3534 | 8.4 | 8.8 | 0.2%  (-1.2%, 1.7%) | **-2.2%**  **(-6.7%, 2.4%)** |
| Bunif2 |  |  |  |  |  |  |  |
| 0-1 | <30 | 29 | 414 | 7.0 | 5.0 | Ref |  |
|  | ≥30 | 22 | 162 | 13.6 | 12.3 | 7.3%  (0.3%, 14.3%) |  |
| ≥ 2 | <30 | 602 | 7485 | 8.0 | 8.4 | Ref |  |
|  | ≥30 | 304 | 3629 | 8.4 | 8.6 | 0.2%  (-1.2%, 1.6%) | **-7.1%**  **(-14.2%, 0.0%)** |

^a^ Adjusted for beach, age, mean bathers, sand, rain, water temperature

**Table S6.** Continued

| **Diarrhea** | | | | | | | |
| --- | --- | --- | --- | --- | --- | --- | --- |
| Marker (samples) | Enterococcus (CFU/100ml) | Cases | N | Crude Risk (%) | Adjusted Risk (%)^a^ | Adjusted  RD (95% CI) ^a^ | Interaction Contrast  (95% CI) |
| -- | Main association |  |  |  |  | 0.7%  (-0.4%, 1.9%) |  |
| HumM |  |  |  |  |  |  |  |
| 0-1 | <30 | 248 | 4316 | 5.7 | 5.5 | Ref |  |
|  | ≥30 | 104 | 1398 | 7.4 | 6.7 | 1.2%  (-0.5%, 3.0%) |  |
| ≥ 2 | <30 | 168 | 3583 | 4.7 | 5.2 | Ref |  |
|  | ≥30 | 119 | 2393 | 5.0 | 5.8 | 0.5%  (-1.0%, 2.0%) | **-0.7%**  **(-3.0%, 1.6%)** |
| HF183 |  |  |  |  |  |  |  |
| 0-1 | <30 | 130 | 1888 | 6.9 | 5.5 | Ref |  |
|  | ≥30 | 60 | 617 | 9.7 | 7.1 | 1.6%  (-0.9%, 4.2%) |  |
| ≥ 2 | <30 | 286 | 6011 | 4.8 | 5.4 | Ref |  |
|  | ≥30 | 163 | 3174 | 5.1 | 5.8 | 0.5%  (-0.8%, 1.7%) | **-1.2%**  **(-4.0%, 1.7%)** |
| BsteriF1 |  |  |  |  |  |  |  |
| 0-1 | <30 | 87 | 1611 | 5.4 | 4.4 | Ref |  |
|  | ≥30 | 19 | 257 | 7.4 | 6.1 | 1.7%  (-2.1%, 5.5%) |  |
| ≥ 2 | <30 | 329 | 6288 | 5.2 | 5.7 | Ref |  |
|  | ≥30 | 204 | 3534 | 5.8 | 6.2 | 0.5%  (-0.7%, 1.7%) | **-1.2%**  **(-5.1%, 2.8%)** |
| Bunif2 |  |  |  |  |  |  |  |
| 0-1 | <30 | 21 | 414 | 5.1 | 3.7 | Ref |  |
|  | ≥30 | 17 | 162 | 10.5 | 9.6 | 5.9%  (-0.6%, 12.3%) |  |
| ≥ 2 | <30 | 395 | 7485 | 5.3 | 5.5 | Ref |  |
|  | ≥30 | 206 | 3629 | 5.7 | 6.0 | 0.4%  (-0.7%, 1.6%) | **-5.5%**  **(-12.0%, 1.1%)** |

NA, not able to estimated

^a^ Adjusted for beach, age, mean bathers, sand, rain, water temperature

**Table S6.** Continued

| **Respiratory Illness** | | | | | | | |
| --- | --- | --- | --- | --- | --- | --- | --- |
| Marker (samples) | Enterococcus (CFU/100ml) | Cases | N | Crude Risk (%) | Adjusted Risk (%)^a^ | Adjusted  RD  (95% CI) ^a^ | Interaction Contrast  (95% CI) |
| -- | Main association |  |  |  |  | -0.9%  (-2.0%, 0.2%) |  |
| HumM |  |  |  |  |  |  |  |
| 0-1 | <30 | 263 | 4174 | 6.3 | 6.2 | Ref |  |
|  | ≥30 | 72 | 1371 | 5.3 | 5.0 | -1.2%  (-2.7%, 0.4%) |  |
| ≥ 2 | <30 | 205 | 3475 | 5.9 | 6.4 | Ref |  |
|  | ≥30 | 131 | 2315 | 5.7 | 5.5 | -0.9%  (-2.4%, 0.7%) | **0.3%**  **(-1.9%, 2.4%)** |
| HF183 |  |  |  |  |  |  |  |
| 0-1 | <30 | 123 | 1837 | 6.7 | 5.8 | Ref |  |
|  | ≥30 | 26 | 613 | 4.2 | 3.7 | -2.1%  (-4.3%, 0.0%) |  |
| ≥ 2 | <30 | 345 | 5812 | 5.9 | 6.4 | Ref |  |
|  | ≥30 | 177 | 3073 | 5.8 | 5.7 | -0.7%  (-2.0%, 0.6%) | **1.4%**  **(-1.1%, 4.0%)** |
| BsteriF1 |  |  |  |  |  |  |  |
| 0-1 | <30 | 80 | 1539 | 5.2 | 4.9 | Ref |  |
|  | ≥30 | 19 | 255 | 7.5 | 6.4 | 1.5%  (-2.3%, 5.3%) |  |
| ≥ 2 | <30 | 388 | 6110 | 6.4 | 6.7 | Ref |  |
|  | ≥30 | 184 | 3431 | 5.4 | 5.3 | -1.4%  (-2.6%, -0.2%) | **-2.9%**  **(-6.9%, 1.1%)** |
| Bunif2 |  |  |  |  |  |  |  |
| 0-1 | <30 | 23 | 401 | 5.7 | 5.3 | Ref |  |
|  | ≥30 | 15 | 162 | 9.3 | 8.6 | 3.3%  (-2.9%, 9.6%) |  |
| ≥ 2 | <30 | 445 | 7248 | 6.1 | 6.4 | Ref |  |
|  | ≥30 | 188 | 3524 | 5.3 | 5.2 | -1.2%  (-2.3%, -0.1%) | **-4.5%**  **(-11.0%, 1.9%)** |

NA, not able to estimated

^a^ Adjusted for beach, age, mean bathers, sand, rain

**Table S7 (marine beaches).** Modification of the association between *Enterococcus* (CCE/100ml) exposure^a^ and illness with human-associated *Bacteroides* markers, marine beaches

| Marker (samples) | Adjusted Risk (%) | Adjusted RD (95% CI) | Interaction Contrast (95% CI) |
| --- | --- | --- | --- |
| ***Gastrointestinal Illness***^a^ | | | |
| **--** |  | 1.6% (1.1%, 2.0%) |  |
| **HumM2** |  |  |  |
| 0-1 | 1.3 | Ref |  |
|  | 2.9 | 1.6% (0.9%, 2.3%) |  |
| ≥ 2 | 3.8 | Ref |  |
|  | 4.6 | 0.8% (-4.0%, 5.6%) | **-0.8% (-5.6%, 4.0%)** |
| **HF183** |  |  |  |
| 0-1 | 0.9 | Ref |  |
|  | 2.1 | 1.3% (0.1%, 2.5%) |  |
| ≥ 2 | 2.8 | Ref |  |
|  | 4.5 | 1.6% (0.7%, 2.6%) | **0.4% (-1.2%, 1.9%)** |
| **BsteriF1** |  |  |  |
| 0-1 | 1.5 | Ref |  |
|  | 2.8 | 1.3% (0.7%, 2.0%) |  |
| ≥ 2 | 1.6 | Ref |  |
|  | 3.3 | 1.7% (1.1%, 2.3%) | **0.4% (-0.6%, 1.3%)** |
| **BuniF2** |  |  |  |
| 0-1 | 0.3 | Ref |  |
|  | 1.2 | 0.9% (-0.7%, 2.4%) |  |
| ≥ 2 | 3.0 | Ref |  |
|  | 4.5 | 1.5% (0.3%, 2.7%) | **0.6% (-1.2%, 2.5%)** |
| ***Diarrhea***^b^ | | | |
| **--** |  | 0.9% (0.4%, 1.5%) |  |
| **HumM2** |  |  |  |
| 0-1 | 0.7 | Ref |  |
|  | 1.6 | 1.0% (0.2%, 1.7%) |  |
| ≥ 2 | 1.3 | Ref |  |
|  | 2.0 | 0.7% (-0.4%, 1.8%) | **-0.3% (-1.6%, 1.1%)** |
| **HF183** |  |  |  |
| 0-1 | 0.5 | Ref |  |
|  | 1.4 | 0.9% (-0.3%, 2.1%) |  |
| ≥ 2 | 0.8 | Ref |  |
|  | 1.7 | 0.9% (0.2%, 1.6%) | **0.0% (-1.5%, 1.5%)** |
| **BsteriF1** |  |  |  |
| 0-1 | 0.9 | Ref |  |
|  | 1.8 | 0.9% (0.2%, 1.6%) |  |
| ≥ 2 | 0.6 | Ref |  |
|  | 1.6 | 1.0% (0.2%, 1.7%) | **0.1% (-1.0%, 1.1%)** |
| **BuniF2** |  |  |  |
| 0-1 | 0.3 | Ref |  |
|  | 1.1 | 0.8% (-0.7%, 2.3%) |  |
| ≥ 2 | 0.7 | Ref |  |
|  | 1.6 | 0.9% (0.2%, 1.6%) | **0.1% (-1.6%, 1.8%)** |
| ***Respiratory Illness*** ^c^ | | | |
| **--** |  | 1.2% (0.9%, 1.6%) |  |
| HumM2 |  |  |  |
| 0-1 | 0.9 | Ref |  |
|  | 2.1 | 1.2% (0.5%, 2.0%) |  |
| ≥ 2 | 10.6 | Ref |  |
|  | 7.1 | -3.5% (-32.2%, 25.3%) | **-4.7% (-33.5%, 24.1%)** |
| HF183 |  |  |  |
| 0-1 | 2.0 | Ref |  |
|  | 3.1 | 1.1% (0.1%, 2.1%) |  |
| ≥ 2 | 1.2 | Ref |  |
|  | 2.5 | 1.3% (0.6%, 2.1%) | **0.2% (-1.2%, 1.6%)** |
| BsteriF1 |  |  |  |
| 0-1 | 0.9 | Ref |  |
|  | 1.9 | 1.0% (-0.1%, 2.1%) |  |
| ≥ 2 | 1.6 | Ref |  |
|  | 2.9 | 1.3% (1.0%, 1.7%) | **0.3% (-0.9%, 1.5%)** |
| BuniF2 |  |  |  |
| 0-1 | 3.6 | Ref |  |
|  | 5.8 | 2.2% (0.1%, 4.3%) |  |
| ≥ 2 | 0.5 | Ref |  |
|  | 1.4 | 0.9% (0.1%, 1.7%) | **-1.3% (-3.7%, 1.1%)** |

Abbreviations: CCE, calibrator cell equivalents; CI, confidence interval; RD, risk difference; Ref, reference.

^a^ *Enterococcus* exposure measured by qPCR and coded continuously as an average log_10_ count of *Enterococcus* CCE/100ml per day.

^b^ Adjusted for beach, age, mean bathers, sand, rainfall since 3pm the previous day, water temperature

^c^ Adjusted for beach, age, mean bathers, sand, rainfall since 3pm the previous day

**Table S8 (marine beaches).** Modification of the association between *Enterococcus* (CFU/100ml) exposure^a^ and illness with human-associated *Bacteroides* markers, marine beaches

| Marker (samples) | Adjusted Risk (%) | Adjusted RD (95% CI) | Interaction Contrast (95% CI) |
| --- | --- | --- | --- |
| ***Gastrointestinal Illness***^a^ | | | |
| **--** |  | -1.9% (-5.9%, 2.1%) |  |
| **HumM2** |  |  |  |
| 0-1 | 9.0 | Ref |  |
|  | 7.6 | -1.5% (-6.4%, 3.5%) |  |
| ≥ 2 | 7.8 | Ref |  |
|  | 6.5 | -1.3% (-7.6%, 5.1%) | **0.2% (-6.4%, 6.8%)** |
| **HF183** |  |  |  |
| 0-1 | 7.5 | Ref |  |
|  | 6.4 | -1.1% (-6.4%, 4.3%) |  |
| ≥ 2 | 11.1 | Ref |  |
|  | 8.3 | -2.8% (-8.0%, 2.4%) | **-1.7% (-8.6%, 5.2%)** |
| **BsteriF1** |  |  |  |
| 0-1 | 4.0 | Ref |  |
|  | 5.2 | 1.3% (-1.6%, 4.1%) |  |
| ≥ 2 | 10.2 | Ref |  |
|  | 7.4 | -2.8% (-7.8%, 2.3%) | **-4.0% (-9.3%, 1.2%)** |
| **BuniF2** |  |  |  |
| 0-1 | 1.9 | Ref |  |
|  | 4.0 | 2.1% (0.9%, 3.3%) |  |
| ≥ 2 | 11.2 | Ref |  |
|  | 7.1 | -4.2% (-9.4%, 1.1%) | **-6.3% (-11.3%, -1.2%)** |
| ***Diarrhea***^b^ | | | |
| **--** |  | -1.2% (-4.8%, 2.3%) |  |
| **HumM2** |  |  |  |
| 0-1 | 5.8 | Ref |  |
|  | 5.2 | -0.5% (-4.8%, 3.7%) |  |
| ≥ 2 | 4.4 | Ref |  |
|  | 3.7 | -0.7% (-6.1%, 4.7%) | **-0.2% (-5.9%, 5.6%)** |
| **HF183** |  |  |  |
| 0-1 | 5.8 | Ref |  |
|  | 4.8 | -1.0% (-6.4%, 4.4%) |  |
| ≥ 2 | 6.1 | Ref |  |
|  | 4.8 | -1.4% (-5.6%, 2.8%) | **-0.4% (-6.7%, 6.0%)** |
| **BsteriF1** |  |  |  |
| 0-1 | 2.6 | Ref |  |
|  | 3.5 | 0.9% (-1.2%, 3.1%) |  |
| ≥ 2 | 7.0 | Ref |  |
|  | 4.8 | -2.2% (-6.8%, 2.5%) | **-3.1% (-7.8%, 1.7%)** |
| **BuniF2** |  |  |  |
| 0-1 | 2.0 | Ref |  |
|  | 3.4 | 1.4% (-0.1%, 2.9%) |  |
| ≥ 2 | 7.8 | Ref |  |
|  | 4.3 | -3.5% (-8.6%, 1.5%) | **-5.0% (-9.9%, 0.0%)** |
| ***Respiratory Illness*** ^c^ | | | |
| **--** |  | -0.6% (-3.5%, 2.4%) |  |
| HumM2 |  |  |  |
| 0-1 | 6.2 | Ref |  |
|  | 6.3 | 0.1% (-3.6%, 3.9%) |  |
| ≥ 2 | 4.9 | Ref |  |
|  | 4.7 | -0.2% (-4.4%, 3.9%) | **-0.4% (-5.5%, 4.8%)** |
| HF183 |  |  |  |
| 0-1 | 5.4 | Ref |  |
|  | 5.1 | -0.2% (-4.3%, 3.8%) |  |
| ≥ 2 | 7.5 | Ref |  |
|  | 6.5 | -1.0% (-4.9%, 2.9%) | **-0.7% (-6.1%, 4.6%)** |
| BsteriF1 |  |  |  |
| 0-1 | 4.0 | Ref |  |
|  | 4.8 | 0.8% (-2.6%, 4.1%) |  |
| ≥ 2 | 6.7 | Ref |  |
|  | 6.0 | -0.7% (-4.4%, 3.0%) | **-1.5% (-6.1%, 3.2%)** |
| BuniF2 |  |  |  |
| 0-1 | 10.8 | Ref |  |
|  | 8.9 | -1.9% (-11.0%, 7.1%) |  |
| ≥ 2 | 6.3 | Ref |  |
|  | 4.8 | -1.5% (-5.1%, 2.1%) | **0.4% (-9.4%, 10.3%)** |

Abbreviations: CFU, colony forming units; CI, confidence interval; RD, risk difference; Ref, reference.

^a^ *Enterococcus* exposure measured by culture and coded continuously as an average log_10_ count of *Enterococcus* CFU/100ml per day.

^b^ Adjusted for beach, age, mean bathers, sand, rainfall since 3pm the previous day, water temperature

^c^ Adjusted for beach, age, mean bathers, sand, rainfall since 3pm the previous day

**Table S9a (count, all beaches).** Frequencies and standardized risk differences (95% CI) for the association between illness and number of human-associated Bacteroides markers among body immersion swimmers in all beaches

| **All beaches** | | | | |
| --- | --- | --- | --- | --- |
| **Number of Markers Detected** | **Cases** | **N** | **Adjusted Risk** | **Adjusted RD (95% CI)** |
| **GI** ^a^ |  |  |  |  |
| 0 | 39 | 440 | 6.4% | Ref |
| 1 | 82 | 899 | 7.0% | 0.7% (-2.4%, 3.7%) |
| 2 | 121 | 1044 | 9.3% | 2.9% (-0.2%, 6.1%) |
| 3 | 326 | 4112 | 8.8% | 2.5% (-0.7%, 5.6%) |
| 4 | 389 | 5193 | 8.3% | 1.9% (-1.2%, 5.1%) |
| **Diarrhea** ^a^ |  |  |  |  |
| 0 | 29 | 440 | 5.1% | Ref |
| 1 | 61 | 897 | 4.9% | -0.2% (-2.9%, 2.6%) |
| 2 | 88 | 1044 | 6.7% | 1.7% (-1.1%, 4.4%) |
| 3 | 211 | 4109 | 5.8% | 0.8% (-2.1%, 3.7%) |
| 4 | 250 | 5188 | 5.5% | 0.4% (-2.5%, 3.3%) |
| **Respiratory** ^b^ |  |  |  |  |
| 0 | 26 | 430 | 4.2% | Ref |
| 1 | 52 | 868 | 4.7% | 0.5% (-2.0%, 3.0%) |
| 2 | 58 | 1029 | 4.4% | 0.1% (-2.2%, 2.5%) |
| 3 | 245 | 3964 | 6.9% | 2.7% (0.1%, 5.2%) |
| 4 | 290 | 5042 | 6.2% | 2.0% (-0.6%, 4.5%) |

^a^ Adjusted for beach, age, mean bathers, sand, rain, water temperature

^b^ Adjusted for beach, age, mean bathers, sand, rain

**Table S9b (count, freshwater beaches).** Frequencies and standardized risk differences (95% CI) for the association between illness and number of human-associated *Bacteroides* markers among body immersion swimmers in fresh water beaches

| **Fresh water beaches** | | | | |
| --- | --- | --- | --- | --- |
| **Number of Markers Detected** | **Cases** | **N** | **Adjusted Risk** | **Adjusted RD (95% CI)** |
| **GI** ^a^ |  |  |  |  |
| 0 | 19 | 208 | 6.3% | Ref |
| 1 | 57 | 576 | 7.1% | 0.8% (-3.4%, 5.0%) |
| 2 | 91 | 765 | 9.7% | 3.4% (-0.6%, 7.4%) |
| 3 | 295 | 3683 | 8.9% | 2.7% (-1.3%, 6.7%) |
| 4 | 357 | 4615 | 8.4% | 2.1% (-1.8%, 6.1%) |
| **Diarrhea** ^a^ |  |  |  |  |
| 0 | 13 | 208 | 4.4% | Ref |
| 1 | 44 | 574 | 5.1% | 0.7% (-3.0%, 4.3%) |
| 2 | 69 | 765 | 7.0% | 2.6% (-0.9%, 6.1%) |
| 3 | 192 | 3680 | 6.0% | 1.6% (-1.9%, 5.0%) |
| 4 | 229 | 4610 | 5.6% | 1.3% (-2.2%, 4.7%) |
| **Respiratory** ^b^ |  |  |  |  |
| 0 | 7 | 201 | 2.8% | Ref |
| 1 | 35 | 556 | 4.9% | 2.2% (-0.6%, 4.9%) |
| 2 | 37 | 744 | 3.9% | 1.1% (-1.3%, 3.5%) |
| 3 | 219 | 3545 | 6.8% | 4.0% (1.5%, 6.5%) |
| 4 | 267 | 4470 | 6.2% | 3.4% (1.0%, 5.9%) |

^a^ Adjusted for beach, age, mean bathers, sand, rain, water temperature

^b^ Adjusted for beach, age, mean bathers, sand, rain

**Table S9c (count, marine beaches).** Frequencies and standardized risk differences (95% CI) for the association between illness and number of human-associated *Bacteroides* markers among body immersion swimmers in marine beaches

| **Marine beaches** | | | | |
| --- | --- | --- | --- | --- |
| **Number of Markers Detected** | **Cases** | **N** | **Adjusted Risk** | **Adjusted RD (95% CI)** |
| **GI** ^a^ |  |  |  |  |
| 0 | 20 | 232 | 6.5% | Ref |
| 1 | 25 | 323 | 6.9% | 0.4% (-4.3%, 5.2%) |
| 2 | 30 | 279 | 9.1% | 2.7% (-3.1%, 8.5%) |
| 3 | 31 | 429 | 7.9% | 1.4% (-4.1%, 6.9%) |
| 4 | 32 | 578 | 6.8% | 0.3% (-6.3%, 6.9%) |
| **Diarrhea** ^a^ |  |  |  |  |
| 0 | 16 | 232 | 6.1% | Ref |
| 1 | 17 | 323 | 4.8% | -1.3% (-6.1%, 3.5%) |
| 2 | 19 | 279 | 7.2% | 1.2% (-4.5%, 6.8%) |
| 3 | 19 | 429 | 4.6% | -1.5% (-7.1%, 4.2%) |
| 4 | 21 | 578 | 3.3% | -2.8% (-9.2%, 3.6%) |
| **Respiratory** ^b^ |  |  |  |  |
| 0 | 19 | 229 | 6.9% | Ref |
| 1 | 17 | 312 | 5.4% | -1.5% (-6.8%, 3.7%) |
| 2 | 21 | 285 | 5.9% | -1.0% (-6.5%, 4.4%) |
| 3 | 26 | 419 | 6.6% | -0.3% (-6.5%, 5.8%) |
| 4 | 23 | 572 | 4.8% | -2.1% (-8.5%, 4.2%) |

^a^ Adjusted for beach, age, mean bathers, sand, rain, water temperature

^b^ Adjusted for beach, age, mean bathers, sand, rain

**Figure S1 (0 vs. present in ≥1 samples).** Standardized risk differences and 95% confidence intervals for the association between illness and human-associated *Bacteroides* markers among body immersion swimmers in all, freshwater, and marine beaches. Human-associated indicators categorized as absent vs. present in ≥1 samples/day


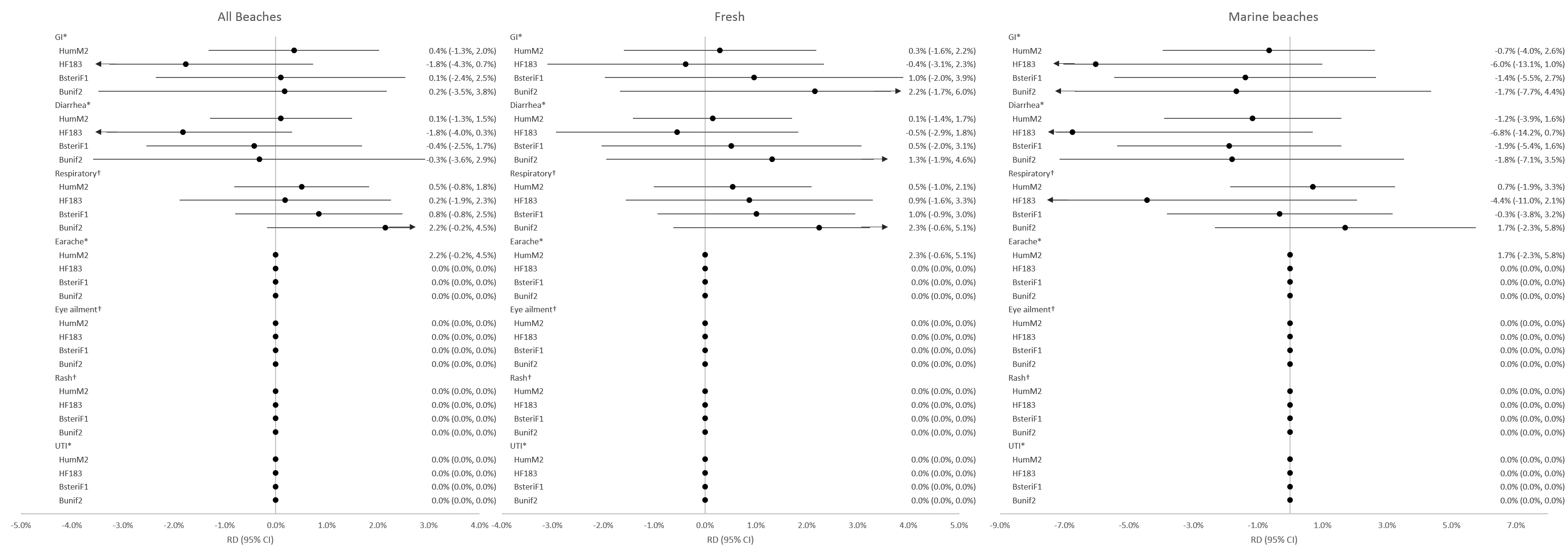

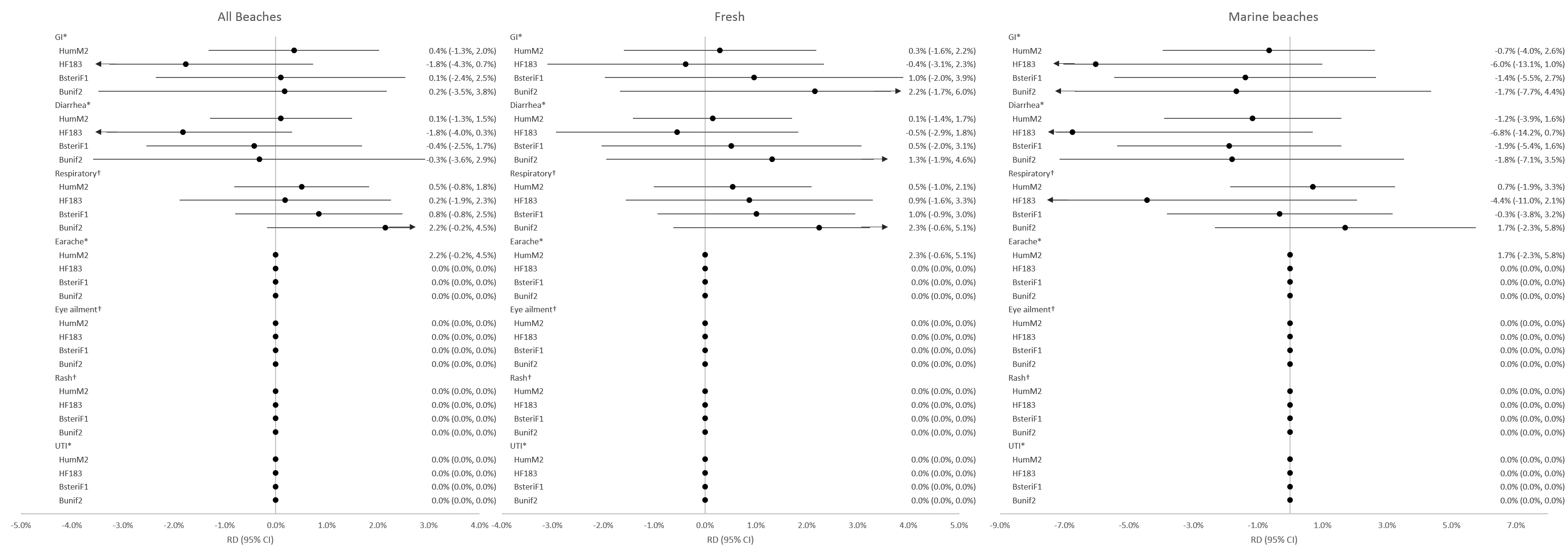


**Table S10 (head immersion).** Frequencies and standardized risk differences (95% CI) for the association between illness and human-associated *Bacteroides* markers among head immersion swimmers in all beaches

|  |  | **All beaches** | | | | |
| --- | --- | --- | --- | --- | --- | --- |
|  |  | **Cases** | **N** | **Crude Risk** | **Adjusted Risk** | **Adjusted RD (95% CI)** |
| **GI** ^a^ |  |  |  |  |  |  |
| HumM2 | 0-1 samples | 365 | 4115 | 8.9% | 8.4% | Ref |
|  | ≥2 samples | 301 | 4230 | 7.1% | 7.9% | -0.6% (-2.1%, 1.0%) |
| HF183 | 0-1 samples | 173 | 1776 | 9.7% | 7.1% | Ref |
|  | ≥2 samples | 493 | 6569 | 7.5% | 8.6% | 1.6% (-0.4%, 3.5%) |
| BsteriF1 | 0-1 samples | 111 | 1328 | 8.4% | 6.6% | Ref |
|  | ≥2 samples | 555 | 7017 | 7.9% | 8.5% | 1.9% (-0.1%, 3.9%) |
| Bunif2 | 0-1 samples | 39 | 424 | 9.2% | 6.8% | Ref |
|  | ≥2 samples | 627 | 7921 | 7.9% | 8.3% | 1.4% (-1.7%, 4.6%) |
| **Diarrhea** ^a^ |  |  |  |  |  |  |
| HumM2 | 0-1 samples | 250 | 4112 | 6.1% | 5.7% | Ref |
|  | ≥2 samples | 180 | 4227 | 4.3% | 4.9% | -0.8% (-2.1%, 0.5%) |
| HF183 | 0-1 samples | 128 | 1774 | 7.2% | 5.2% | Ref |
|  | ≥2 samples | 302 | 6565 | 4.6% | 5.4% | 0.3% (-1.4%, 2.0%) |
| BsteriF1 | 0-1 samples | 73 | 1326 | 5.5% | 4.6% | Ref |
|  | ≥2 samples | 357 | 7013 | 5.1% | 5.5% | 1.0% (-0.6%, 2.6%) |
| Bunif2 | 0-1 samples | 30 | 424 | 7.1% | 5.6% | Ref |
|  | ≥2 samples | 400 | 7915 | 5.1% | 5.4% | -0.3% (-3.2%, 2.6%) |
| **Respiratory** ^b^ |  |  |  |  |  |  |
| HumM2 | 0-1 samples | 241 | 4015 | 6.0% | 5.9% | Ref |
|  | ≥2 samples | 246 | 4092 | 6.0% | 6.3% | 0.4% (-1.0%, 1.7%) |
| HF183 | 0-1 samples | 107 | 1745 | 6.1% | 5.4% | Ref |
|  | ≥2 samples | 380 | 6362 | 6.0% | 6.3% | 0.9% (-1.2%, 2.9%) |
| BsteriF1 | 0-1 samples | 71 | 1273 | 5.6% | 5.0% | Ref |
|  | ≥2 samples | 416 | 6834 | 6.1% | 6.3% | 1.3% (-0.3%, 2.9%) |
| Bunif2 | 0-1 samples | 28 | 413 | 6.8% | 6.1% | Ref |
|  | ≥2 samples | 459 | 7694 | 6.0% | 6.1% | 0.0% (-3.0%, 3.0%) |

^a^ Adjusted for beach, age, mean bathers, sand, rain, water temperature

^b^ Adjusted for beach, age, mean bathers, sand, rain

**Table S11 (swallowed water).** Frequencies and standardized risk differences (95% CI) for the association between illness and human-associated *Bacteroides* markers among swimmers who swallowed water in all beaches

|  |  | **All beaches** | | | | |
| --- | --- | --- | --- | --- | --- | --- |
|  |  | **Cases** | **N** | **Crude Risk** | **Adjusted Risk** | **Adjusted RD (95% CI)** |
| **GI** ^a^ |  |  |  |  |  |  |
| HumM2 | 0-1 samples | 101 | 1009 | 10.0% | 9.4% | Ref |
|  | ≥2 samples | 99 | 1076 | 9.2% | 10.2% | 0.8% (-2.7%, 4.2%) |
| HF183 | 0-1 samples | 52 | 493 | 10.5% | 9.2% | Ref |
|  | ≥2 samples | 148 | 1592 | 9.3% | 10.0% | 0.8% (-3.5%, 5.2%) |
| BsteriF1 | 0-1 samples | 34 | 341 | 10.0% | 8.1% | Ref |
|  | ≥2 samples | 166 | 1744 | 9.5% | 10.1% | 2.0% (-2.4%, 6.5%) |
| Bunif2 | 0-1 samples | 17 | 140 | 12.1% | 12.7% | Ref |
|  | ≥2 samples | 183 | 1945 | 9.4% | 9.6% | -3.2% (-12%, 5.7%) |
| **Diarrhea** ^a^ |  |  |  |  |  |  |
| HumM2 | 0-1 samples | 75 | 1008 | 7.4% | 7.1% | Ref |
|  | ≥2 samples | 60 | 1076 | 5.6% | 6.1% | -1.0% (-3.8%, 1.8%) |
| HF183 | 0-1 samples | 39 | 493 | 7.9% | 7.1% | Ref |
|  | ≥2 samples | 96 | 1591 | 6.0% | 6.5% | -0.6% (-4.4%, 3.3%) |
| BsteriF1 | 0-1 samples | 24 | 341 | 7.0% | 5.4% | Ref |
|  | ≥2 samples | 111 | 1743 | 6.4% | 6.9% | 1.5% (-2.0%, 5.1%) |
| Bunif2 | 0-1 samples | 13 | 140 | 9.3% | 9.3% | Ref |
|  | ≥2 samples | 122 | 1944 | 6.3% | 6.4% | -2.8% (-10.4%, 4.7%) |
| **Respiratory** ^b^ |  |  |  |  |  |  |
| HumM2 | 0-1 samples | 79 | 981 | 8.1% | 7.4% | Ref |
|  | ≥2 samples | 88 | 1029 | 8.6% | 9.3% | 1.9% (-1.4%, 5.2%) |
| HF183 | 0-1 samples | 40 | 481 | 8.3% | 6.4% | Ref |
|  | ≥2 samples | 127 | 1529 | 8.3% | 9.1% | 2.7% (-1.8%, 7.2%) |
| BsteriF1 | 0-1 samples | 28 | 319 | 8.8% | 7.9% | Ref |
|  | ≥2 samples | 139 | 1691 | 8.2% | 8.4% | 0.5% (-3.2%, 4.3%) |
| Bunif2 | 0-1 samples | 17 | 133 | 12.8% | 14.4% | Ref |
|  | ≥2 samples | 150 | 1877 | 8.0% | 7.9% | -6.5% (-17.3%, 4.3%) |

^a^ Adjusted for beach, age, mean bathers, sand, rain, water temperature

^b^ Adjusted for beach, age, mean bathers, sand, rain

**Bibliography**

1. Haugland RA, Varma M, Sivaganesan M, Kelty C, Peed L, Shanks OC. Evaluation of genetic markers from the 16S rRNA gene V2 region for use in quantitative detection of selected Bacteroidales species and human fecal waste by qPCR. Syst Appl Microbiol. 2010;33:348-57.

2. Wade TJ, Sams E, Brenner KP, Haugland R, Chern E, Beach M, et al. Rapidly measured indicators of recreational water quality and swimming-associated illness at marine beaches: a prospective cohort study. Environ Health. 2010;9:66.

3. Siefring S, Varma M, Atikovic E, Wymer L, Haugland RA. Improved real-time PCR assays for the detection of fecal indicator bacteria in surface waters with different instrument and reagent systems. J Water Health. 2008;6:225-37.

4. Shanks OC, Kelty CA, Sivaganesan M, Varma M, Haugland RA. Quantitative PCR for genetic markers of human fecal pollution. Appl Environ Microbiol. 2009;75:5507-13.
